# Laser maskless fast patterning for multitype microsupercapacitors

**DOI:** 10.1038/s41467-023-39760-3

**Published:** 2023-07-05

**Authors:** Yongjiu Yuan, Xin Li, Lan Jiang, Misheng Liang, Xueqiang Zhang, Shouyu Wu, Junrui Wu, Mengyao Tian, Yang Zhao, Liangti Qu

**Affiliations:** 1 Laser Micro/Nano-Fabrication Laboratory, School of Mechanical Engineering, Beijing Institute of Technology, Beijing, PR China; 2 Yangtze Delta Region Academy of Beijing Institute of Technology, Jiaxing, PR China; 3 Beijing Institute of Technology Chongqing Innovation Center, Chongqing, PR China; 4 Department of Mechanical Engineering, City University of Hong Kong, Hong Kong, PR China; 5 School of Instrument Science and Opto-Electronics Engineering, Beijing Information Science and Technology University, Beijing, PR China; 6 Key Laboratory of Cluster Science Ministry of Education of China, School of Chemistry and Chemical Engineering, Beijing Institute of Technology, Beijing, PR China; 7 MOE Key Laboratory of Bioorganic Phosphorus Chemistry & Chemical Biology, Department of Chemistry, Tsinghua University, Beijing, PR China

**Keywords:** Supercapacitors, Electrical and electronic engineering, Electrochemistry

## Abstract

Downsizing electrode architectures have significant potential for microscale energy storage devices. Asymmetric micro-supercapacitors play an essential role in various applications due to their high voltage window and energy density. However, efficient production and sophisticated miniaturization of asymmetric micro-supercapacitors remains challenging. Here, we develop a maskless ultrafast fabrication of multitype micron-sized (10 × 10 μm^2^) micro-supercapacitors via temporally and spatially shaped femtosecond laser. MXene/1T-MoS_2_ can be integrated with laser-induced MXene-derived TiO_2_ and 1T-MoS_2_-derived MoO_3_ to generate over 6,000 symmetric micro-supercapacitors or 3,000 asymmetric micro-supercapacitors with high-resolution (200 nm) per minute. The asymmetric micro-supercapacitors can be integrated with other micro devices, thanks to the ultrahigh specific capacitance (220 mF cm^−2^ and 1101 F cm^−3^), voltage windows in series (52 V), energy density (0.495 Wh cm^−3^) and power density (28 kW cm^−3^). Our approach enables the industrial manufacturing of multitype micro-supercapacitors and improves the feasibility and flexibility of micro-supercapacitors in practical applications.

## Abstract (web summary)

Miniature asymmetric supercapacitors have higher voltage and energy density but are often limited by a complex manufacturing process and difficulties in further miniaturization. Here, the authors demonstrate a maskless method for the patterned fabrication of submicron-scale symmetric and asymmetric supercapacitors.

## Introduction

The burgeoning need for miniaturized, multifunctional portable electronics has triggered significant growth in the field of micro-integrated energy systems. The micro-supercapacitor (MSC) is a core component in microscale energy storage devices^1^. Multiple types of MSCs have been developed, owing to rapid advances in technology. Asymmetric MSCs, unlike symmetric MSCs, can be assembled using two-electrode materials, which enhances their voltage window and results in a substantial increase in energy density^2,3^. Therefore, MSCs may have broad practical applications.

In recent years, noteworthy advancements have been achieved in the development of asymmetric MSCs. Conventional asymmetric MSCs are referred to as sandwich-type MSCs. Thinner, smaller, more flexible planar asymmetric MSCs necessitate many fabrication steps and are difficult to further downsize^4,5^. Specifically, the preparation process is exceedingly complex due to the unique structure of inconsistent electrode materials; besides, accurate management of the electrode materials is difficult. The minimum size of asymmetric supercapacitors remains at the millimeter level. Attaining accurate manipulation of electrode material assembly and concurrent patterning of two distinct material types on a single plane through conventional techniques such as electrodeposition^6^, inkjet printing^7^, laser etching^8^, and photolithography^9^ presents inherent challenges. The femtosecond laser has been used as an in situ efficient machining method during the fabrication of ultra-precision devices^10–12^. Meanwhile, the fabrication of 3D MSCs has been severally achieved by optimizing its unique processing benefits in material structure^13–15^. Pseudocapacitor materials are common asymmetric electrode materials. However, their power density is low and their lifespan is short due to instability and poor electrical conductivity. Two-dimensional materials have attracted considerable research attention due to their excellent electrochemical properties. 1T-MoS_2_ and MXene have highly reversible surface redox reactions and favorable metallic conductivity^16,17^. A recent study revealed that 1T-MoS_2_ has excellent conductivity and high cycle life as an electrode material for asymmetric supercapacitors with a large voltage window^18^. In other studies, MXene was integrated with various metal oxides and carbon-based materials to construct asymmetric supercapacitors, achieving excellent electrochemical performance^19,20^.

In this work, we present a method for the ultrafast fabrication of submicron-scale symmetric and asymmetric MSCs on the 1T-MoS_2_/MXene thin films using temporally and spatially shaped femtosecond laser (TSSF) patterning. We fabricate three types of MSCs, including a 1T-MoS_2_/MXene symmetric MSC, a laser-induced symmetric MSC prepared on MXene-derived TiO_2_ and 1T-MoS_2_-derived MoO_3_ thin films, and an asymmetric MSC prepared on 1T-MoS_2_/MXene/laser-induced MXene-derived TiO_2_ and 1T-MoS_2_-derived MoO_3_ thin films. TSSF technology can control the synthesis of materials by temporal laser pulse shaping, and can realize the micro-nano machining of any shape in one step through the spatial multi-phase stacking patterning. Laser pulse delay of the temporally shaped laser subpulse and energy are used to manipulate the composition of laser-induced MoO_3_ and TiO_2_ thin films. Using the proposed approach, more than 6000 MSCs can be fabricated every minute, each with a size of only 10 × 10 μm^2^. The minimum line width of the MSCs is only 200 nm. As expected, the asymmetric MSCs generate large voltage windows (1.8 V), excellent specific capacitance (220 mF cm^−2^ and 1101 F cm^−3^), and high energy density (0.495 Wh cm^−3^). Notably, the asymmetric MSCs exhibit excellent electrochemical performance across a wide range of scan rates (1 mV s^−1^–500 V s^−1^), achieving a peak power density of 28 kW cm^−3^. Furthermore, they can be flexibly attached to various substrates, including flexible substrates, and demonstrated a long cycle life (98.3% capacitance retention after 15,000 cycles) and excellent mechanical flexibility. We believe that our technology can achieve ultrahigh-speed processing of micro-multi-type supercapacitors, providing a foundation for rapid industrial and mass production. The present strategy also promotes the miniaturization and practical application of asymmetric MSCs, hence providing a new direction in the development of multitype microscale energy storage devices in scalable manufacturing.

## Results

### The Preparation of Multitype MSCs

**Fig. 1:**
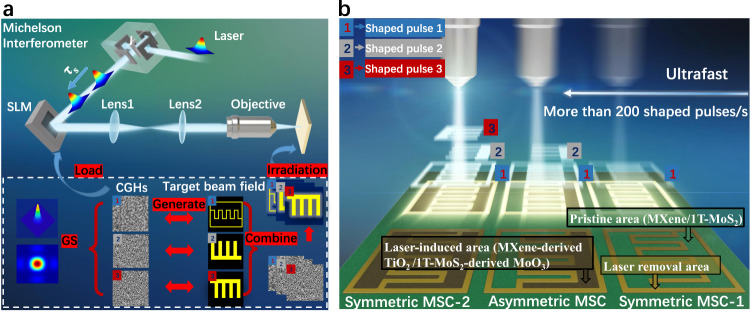
Schematic diagram of the SLM-based maskless patterning method for ultrafast manufacturing of multitype MSCs. **a** The original Gaussian laser is transformed by the Michelson interferometer into a double pulse with a pulse delay. It then passes through the SLM and is transported to the objective lens by the 4f system to realize micro/nano processing. **b** The magnified image of the objective lens and the sample can be processed within an extremely short period by controlling the 1, 2, and 3 subpulses to obtain various types of MSCs.

First, 1T-MoS_2_/MXene hybrid thin films of different thickness were prepared through vacuum filtration. We transferred thin films of different thicknesses to the glass substrate. 1T-MoS_2_/MXene hybrid films of different thicknesses were obtained by controlling the amount of mixed two-dimensional material solution by vacuum filtration, as shown in Supplementary Fig. 1. The scanning electron micrographs (SEM) image of the prepared films clearly shows that the thickness of different films is 1, 2, 5, 8 microns. Supplementary Fig. 2 shows the magnified SEM image of cross-section view of the hybrid film with a thickness of 1 micron and the roughness of the surface. The results show that the surface of the hybrid film is smooth and the roughness is in nanometer level. As shown in Fig. 1a, a confocal temporal and spatial pulse shaping system for femtosecond lasers were constructed to achieve ultrafast, high-precision patterning. Prior to entering into the Spatial Light Modulator (SLM), the Gaussian laser beam was subjected to focalization by means of a Michelson interferometer. Subsequently, the Gaussian femtosecond pulse was incorporated into a bipartite pulse train that was uniformly divided, featuring a pulse delay of 10 picoseconds (ps).

Notably, various types of symmetric and asymmetric MSCs can be fabricated by designing any combination of light fields. TSSL can control the synthesis of materials through the temporally shaped laser pulses, and can realize the micro-nano processing of any shape in one step through the spatial multi-phase stacking patterning. Benefiting from the femtosecond laser’s uniquely ultrahigh peak power (>10^13^ W cm^−2^) and ultrashort irradiation period, the strategy can process nearly any material^21^. Phase-adjustable SLM was adopted to shape the initial Gaussian femtosecond laser (800 nm, 35 fs) into a femtosecond laser with a varying spatial distribution. By designing different phases, arbitrary changes in the light field were achieved within an extremely short period to produce multiple types of MSCs. Of note, SLM can load computer-generated holograms (CGHs) to shape light in space^22^. In previous experiments using SLM, each pattern corresponded to a CGH^23,24^. However, considering the processing pattern of multiple types of MSCs, various patterns must be simultaneously processed in the asymmetric MSCs in situ.

In this study, the target pattern was superimposed on multiple target images. To achieve continuous changes in multiple light fields, programming techniques were employed to load various CGHs onto the SLM. Through the utilization of a 4f relay system, the SLM outlet produced several spatially focused light fields, which were subsequently smoothly focused by the objective lens. As shown in Supplementary Fig. 3, the original material (1T-MoS_2_/MXene) was irradiated with different-shaped pulse lasers, and shaped light field 1 was used to remove the 1T-MoS_2_/MXene. Further, light fields 2 and 3 were shaped to modify the material in order to obtain laser-induced MXene-derived TiO_2_/1T-MoS_2_-derived MoO_3_. Consequently, the whole process realizes two processes laser-induced material synthesis and laser removal. As shown in Fig. 1b, a high-precision symmetric MSC (1T-MoS_2_/MXene) with an adjustable size was fabricated when light field pattern 1 was individually focused and processed on the thin film sample. At this point, ablation was performed via laser focusing using light field pattern 1. By adjusting energy or regulating the number of subpulse repetitions, light field 1 could completely remove the material, whereas light field 2 required laser induction to modify the material. When light field patterns 1 and 2 were integrated to focus on the thin film sample, the asymmetric MSC (1T-MoS_2_/MXene//laser-induced MXene-derived TiO_2_ /1T-MoS_2_-derived MoO_3_) was obtained with similar precision. The simultaneous projection of light fields 1, 2, and 3 yielded another symmetric MSCs (laser-induced MXene-derived TiO_2_ and 1T-MoS_2_-derived MoO_3_). In this process, materials were subjected to laser ablation based on laser energy. We could accurately control the power of laser processing using optical instruments to achieve different approaches to processing materials. As shown in Supplementary Fig. 4, we precisely regulated the laser power to selectively eliminate and modify the material combined with the actual processing effect of the hybrid film.

**Fig. 2:**
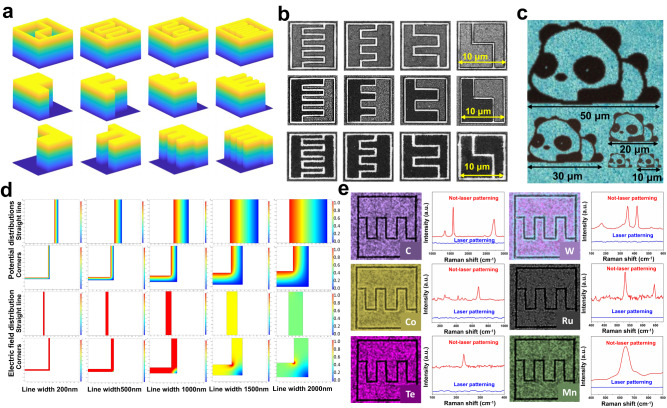
The results of TSSF patterning, simulation and applicability of multi-material systems. **a** Light field patterns of differing shapes. **b** Optical micrographs of various types of MSCs obtained through processing (scale bars: 10 μm). **c** Scanning electron micrographs of MSCs of different sizes. **d** The simulated potential and electric field distributions of micro-supercapacitors with different line widths at straight lines and corners by the COMSOL soft. **e** Scanning electron microscopy mapping of patterns on different materials and the corresponding Raman spectra.

As presented in Fig. 2a, the patterns of the target light field obtained were different and could be arbitrarily combined to fabricate multiple types of MSCs. As previously mentioned, our technology is capable of processing MSCs of different types with a minimum size of 10 × 10 μm^2^ (as shown in Fig. 2b). Moreover, in order to meet the personalized size requirements of different micro-nano devices for energy storage devices, our technology can also expand to process larger and personalized micro-patterns, as shown in Fig. 2c, and the size of the panda patterns can be arbitrarily adjusted. Moreover, MSCs of different personalized patterns can be prepared by controlling the shapes of the target light field (Supplementary Fig. 5).

Our technology promotes the fabrication of asymmetrical micro-supercapacitors with the smallest size and highest resolution. In micro-supercapacitors, the narrow gap may have a significant effect on device performance, and mediate charge ion transfer between electrode materials and electrolytes. As shown in Supplementary Fig. 6, we prepared micro-supercapacitors with different narrow gaps; the narrow gap can be reduced from 5 μm to 200 nm. The SEM magnification shows that the width of the narrow gap at three selected positions is 203 nm, confirming the good uniformity of our processing technology. As shown in Supplementary Fig. 7a, b, element mapping of Mo and Ti was performed on two laser-patterned MSCs; their partial magnification is shown in c and d. As shown, the edges of the machining are fairly regular with no obvious detrital deposition. To further investigate the effect of the narrow gap width on electrochemical performance, we performed corresponding simulations of potential and electric fields by COMSOL at straight lines and corners of the narrow gaps. Figure 2d shows the potential distribution and electric field distribution of micro-supercapacitors with different widths (200, 500, 1000, 1500, and 2000 nm) of the narrow gaps. Subsequently, we conducted simulation statistics on the distribution curve of potential (Supplementary Fig. 8) and electric field (Supplementary Fig. 9). When the line width was 2 μm, the unidirectional potential was simulated in the range of 0.2~0.8 V. When the line width was 200 nm, the simulated potential distribution was approximately 0.35–0.65 V. On the other hand, the smaller the line width, the higher the electric field distribution intensity in the electric field distribution. The electric field intensity of a micro-supercapacitor with a narrow gap of 200 nm was 2.8 ~ 5 × 10^6^ V/m, much higher than that of a micro-supercapacitor with a narrow gap of 2 μm (0.3~0.5 × 10^6^ V/m). Therefore, the difference in narrow gap width causes the potential difference and electric field distribution. To evaluate the impact of interdigital gap width (200 nm, 500 nm, 1 μm, and 2 μm) on electrochemical performance, cyclic voltammograms (CVs) were measured (Supplementary Fig. 10). The differences of areal capacitance were observed in MSCs with varying narrow gaps. The energy storage mechanism of the prepared asymmetric MSCs comprises of both the electric double-layer capacitor (EDLC) energy storage mechanism and Faraday effect. The narrow gap, which possesses extremely high precision, plays a crucial role in facilitating ion transport, as well as the pseudo capacitance reaction of the material. The material experiences changes during this process, which may further be influenced by the narrow gap. It should be noted that simply reducing the width of the narrow gap does not guarantee a continuous improvement in the performance of the MSCs. Our experimental findings reveal that the maximum capacitance value was obtained at a narrow gap of 500 nm (Supplementary Fig. 11). Therefore, we can infer that the narrow gap of 500 nm in this work is a suitable value for the performance of the entire device. On the other hand, we also prepared micro-supercapacitors with different thicknesses and evaluated their electrochemical properties (Supplementary Figs. 12 and 13). As expected, the thicker the micro-supercapacitor, the higher its areal capacitance and the lower its volumetric capacitance.

Moreover, we could regulate the laser frequency in the actual processing procedure; laser pulses could be extremely and rapidly shot out of the laser. In the processing of MSCs with a size of 10 × 10 μm^2^, the translation was set to move at 2000 μm s^−1^ under a laser frequency of 200 Hz. Therefore, 200 subpulses could be used to theoretically pattern 200 symmetric MSCs in 1 s. Given the stability of the translation stage and the spacing between symmetric MSCs in the actual processing, the preparation of 6000 MSCs per minute was achieved. However, since many patterned light fields are necessary to realize the fabrication of an asymmetric MSC, 3000 is the maximum number of asymmetric MSCs processed per minute. Supplementary movies 1 and 2 show TSSF ultrafast processing of symmetric and asymmetric micro-supercapacitors and the size of each MSC was 10 × 10 μm^2^. Femtosecond laser pulses were utilized to realize pattern processing of various electrode material systems since this technology can be used to process nearly any material. As shown in Fig. 2e, regular pattern processing was performed on various materials (a metal-organic framework, graphene, WS_2_, MoTe_2_, MnO_2_, and RuO_2_). Raman characterization of the laser-patterned area confirmed the complete removal of the material via laser ablation. This indicates that our technology can be applied to the pattern processing of two-dimensional materials and the high-precision processing of metal oxide materials. The present study provides promising findings regarding the applicability of our technology in the fabrication of microelectronics and microscale energy storage devices. The investigation included a thorough review of the technologies used in processing asymmetric supercapacitors, which culminated in a comparison of our technology with other existing processes. The comparative analysis is presented in Supplementary Table 1, which highlights the superiority of our technology in terms of size and maximum resolution of the planar asymmetric supercapacitors produced. Specifically, our technology enables the production of asymmetric MSCs at a micron scale, which is significantly smaller than those fabricated using conventional methods. Moreover, our technology facilitates a substantial improvement in the processing resolution of asymmetric MSCs. Notably, our TSSF has demonstrated remarkable success in manufacturing and performance enhancement, unlike traditional lasers, with respect to processing technology.

As shown in Supplementary Table 2, we performed a comprehensive comparison of performance, device parameters, and machining efficiency with other laser-machined supercapacitors. Notably, the miniature supercapacitors we prepared are effective in all aspects.

### Characterization and Analysis of Laser-Induced MXene/1T-MoS_2_ Materials

**Fig. 3:**
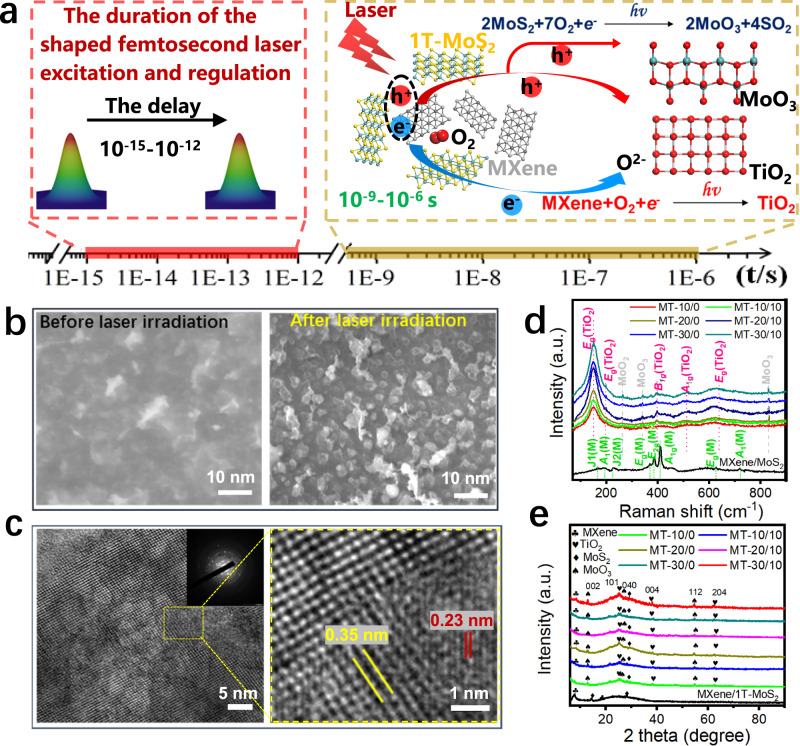
Schematic diagram and characterization of TSSF-induced material synthesis. **a** The schematic diagram of photo-induced chemical synthesis of 1T-MoS_2_/MXene. **b** Scanning electron micrographs of the material surfaces before and after laser irradiation. **c** Transmission electron micrographs (TEM) captured after the laser-induced modification of the material. **d** Raman spectra of each composite materials. **e** X-ray diffraction (XRD) patterns of each composite material.

Figure 3a presents a schematic diagram of photo-induced chemical synthesis of 1T-MoS_2_/MXene two-dimensional composite materials under the action of double-pulse femtosecond laser. The pulse delay of the femtosecond laser (10^−15^–10^−12^ s) is much shorter than the duration of the photochemical/photothermal synthesis of the original material (10^−9^–10^−6^ s). Therefore, this method can reveal the regulation mechanism of selective atomic/electron excitation, chemical reaction path and non-equilibrium heat transport by TSSF. The figure presented indicates that the initial femtosecond pulse laser induced a significant number of free electrons and holes. The subsequent subpulses focused the laser on the same material. When the front sequence pulse contacted the material, numerous freely moving electrons were excited. The subsequent pulse sequence further interacted with the seed electrons generated by the front sequence pulse before the material was modified or ablated, leading to the avalanche ionization of more free electron eruptions. This occurrence is because the pulse delay between the two pulse sequences is in the picosecond order, which is substantially shorter than the time required for material phase transition. With the help of these free electrons, oxygen in the air reacts chemically with the two-dimensional material to form new heterojunctions. The extremely high instantaneous power of the femtosecond laser pulses generated defects in the Mo–S and Ti–C bonds. The abundant free electrons present during the femtosecond laser irradiation process facilitated the interaction between the laser pulses and oxygen molecules in the ambient air. Moreover, the femtosecond laser pulses ionized to the oxygen to produce oxygen bonds. Thus, the original MXene and 1T-MoS_2_ material were easily transformed into metal oxide.

Figure 3b presents SEM images of the surface morphology of the composite material before and after processing. The numerous metal oxide particles generated on the material surface after the shaped laser processing show that oxidation occurred during processing. The scanning electron micrographs in Supplementary Fig. 14a–f indicates that the nanoparticles appeared on the surface of the laser-induced material with increasing laser energy. Moreover, the morphology and size of the nanoparticles underwent regular changes with changing laser energy. The size of these nanoparticles ranged from about 10–30 nm, significantly increasing the specific surface area of the material and providing a huge number of sites for generating laser-induced metal oxides. Notably, the transmission electron micrographs and high-resolution transmission electron micrographs of laser-induced materials (Fig. 3c) validate the presence of TiO_2_ and MoO_3_. The composed materials (MoO_3_/TiO_2_) induced by the focused laser at 10 mW with 0 pulse delay were labeled as MT-10/0. In our experiment, the laser power gradually increased from 10 to 30 mW, and the laser pulse delay distribution was 0 and 10 ps. Supplementary Fig. 15 presents the transmission electron micrographs and selected area electron diffraction patterns of MXene/1T-MoS_2_, MT-10/0, MT-10/10, MT-20/0, MT-20/10, MT-30/0, and MT-30/10. Lattice fringes were observed in the high-resolution transmission electron micrographs, where the d-space of 0.35 nm corresponds to the (101) plane of anatase TiO_2_^25^. In the high-resolution transmission electron micrographs of typical orthogonal MoO_3_ nanorods, lattice fringes were visible. The distance between adjacent fringes was about 0.23 nm, indicating that the nanorods grew in the (200) direction^26^. The transmission electron micrographs revealed that the corresponding metal oxides were formed during the laser ablation of MXene/1T-MoS_2_. The Raman spectra of all samples were analyzed to further assess the content of titanium oxide and molybdenum oxide in the composite materials (Fig. 3d). We observed obvious peaks at 50–900 cm^−1^ corresponding to the distinct vibration modes of the composite materials. Regarding MXene/1T-MoS_2_, the peaks located at 198 and 710 cm^−1^ were related to the *A*_1g_ group vibrations of Ti and C atoms. Notably, the additional strong peaks at 167 (J1), 225 (J2), and 387 (J3) cm^−1^ are consistent with that of 1T-MoS_2_. Moreover, the additional peaks of the *E*_g_ group vibrations of MXene corroborate with that reported in other studies^27,28^. The anatase phase of induced TiO_2_ shows major Raman bands at 150, 198, 401, 515, and 641 cm^−1^. These bands can be attributed to the five Raman-active modes of the anatase phase with the symmetries of *E*_g_, *E*_g_, *B*_1g_, *A*_1g_, and *E*_g_, respectively. The peaks of molybdenum oxide near 263, 342, and 831 cm^−1^ of orthorhombic MoO_3_ compound, can be attributed to the Raman-active modes of *B*_3g_, *B*_1g_, and *A*g^29,30^. The X-ray diffraction patterns of different MT and MXene/1T-MoS_2_ were analyzed (Fig. 3e). The diffraction peaks of MXene/1T-MoS_2_ located at 7.78°, 14.38°, 18.28°, and 28.2° corresponded to the expected diffraction (002) peak of MXene, (002) peak of MoS_2_, (006) peak of MXene, and (004) peak of MoS_2_, respectively^27,31^. The diffraction peaks considerably changed when the focused laser pulses were applied to the material. The characteristic diffraction peaks of the original MXene/1T-MoS_2_ remained. Moreover, many novel characteristic diffraction peaks were detected at 12.86°, 25.27°, 26.90°, 38.18°, 54.69°, and 62.47°. They corresponded to the diffraction peaks characteristic of the (020), (040), and (112) crystal planes of MoO_3_, as well as to that of the (101), (004), (204) crystal planes of anatase TiO_2_, confirming the effective oxidation of Ti and Mo in MXene/1T-MoS_2_ during laser ablation. The distinct peak intensities of the characteristic peaks and the slightly stronger intensity of TM10 may be attributed to the extensive modification of the MXene material under laser pulsing. Based on the Raman and XRD spectra of Fig. 3d, e, we also summarized the content ratio of TiO_2_ and MoO_3_ in photochemical synthesis under different parameters (Supplementary Fig. 16).

### Effects of laser parameters on laser-induced materials

**Fig. 4:**
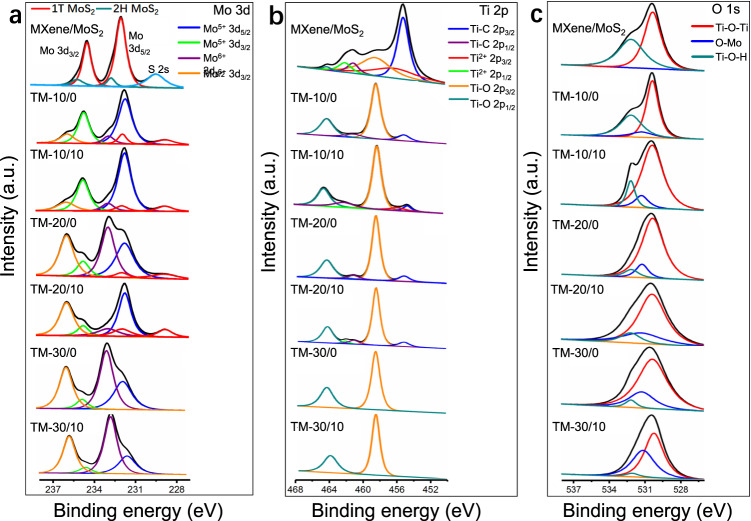
The XPS results of TSSF-induced materials. **a**–**c** Mo 3*d*, Ti 2*p*, and O 1*s* peaks observed in the XPS analysis of composites prepared under various laser energy and laser pulse delay conditions.

X-ray photoelectron spectroscopy (XPS) was conducted to further evaluate the effects of laser parameters on MXene/1T-MoS_2_ after laser ablation. Studies indicate that the binding energy of 1T-MoS_2_ is nearly 0.9 eV lower than that of 2H-MoS_2_ in non-laser–processed materials^25,32^. As shown in Fig. 4a, the high-resolution XPS spectra of Mo 3d could be deconvoluted into peaks assigned to Mo 3*d*_3/2_ and Mo 3*d*_5/2_. The peaks at 231.6, 232.8, 234.6, and 235.8 eV in various MT thin films revealed that the Mo 3*d*_5/2–3/2_ doublets correspond to MoO_3_ and MoS_2_ (with Mo^5+^ 3*d*_5/2_ peaks at 231.6 eV, Mo^5+^ 3*d*_3/2_ peaks at 234.6 eV, Mo^6+^ 3*d*_5/2_ peaks at 232.8 eV, and Mo^5+^ 3*d*_5/2_ peaks at 235.8 eV, respectively)^25,33^. The content of Mo in distinct valence states could be summarized from XPS analysis. The Ti 2*p* spectra confirmed the presence of TiO_2_. The peaks centered at 455.1 and 461.2 eV (Fig. 4b) corresponded to Ti–C bonds. The peaks centered at 458.5 and 464.4 eV were assigned to Ti–O 2*p*_3/2_ and Ti–O 2*p*_1/2_, confirming that the oxygen in TiO_2_ caused the formation of C–Ti–O^34,35^. To comprehensively demonstrate the element composition ratios of MT materials processed under varying laser parameters, we conducted a study using five groups of samples processed under identical conditions. Each group of material samples consisted of all laser-induced MT materials processed under different parameters. Through the statistical analysis of XPS data for each group of materials, we calculated the mean values and standard errors of the proportion of different elements present, to illustrate the distribution of materials. According to the findings presented in Table 1, variations in laser power and pulse delay induced changes in Mo proportion, thereby affirming the potential for modifying the laser parameters to regulate the composition of mixed materials and MoO_3_ content. Additionally, our study provided a summary of the results obtained from the investigation of unprocessed hybrid materials and TM materials processed under different parameters. Notably, the Ti–C bond in MXene underwent a significant reduction following laser ablation, while the Ti–O bond was observed to increase considerably, indicating the production of titanium oxide. Furthermore, the small standard error values obtained for each group of data relative to the mean value suggests that different MT materials possess a stable and similar composition of elements, thus reinforcing the controllability of the synthetic process for MT materials. These findings offer valuable insights into the application of laser ablation for material synthesis and demonstrate its potential for achieving precise and controlled material compositions.

**Table 1:** Summary of the XPS data for the MoS_2_/MXene and different laser-induced TM

Material	Mo^4+^ 3*d*_5/2_ [eV] Mean ± SE%	Mo^5+^ 3*d*_5/2_ [eV] Mean ± SE%	Mo^6+^ 3*d*_5/2_ [eV] Mean ± SE%	Ti–C 2*p* [eV] Mean ± SE%	Ti^2+^ 2*p*_3/2_ [eV] Mean ± SE%	Ti–O 2*p*_3/2_ [eV] Mean ± SE%
MoS_2_/ MXene	228.7/231.8 (1T-86.20 ± 0.75) 229.6/232.5 (2H-12.55 ± 0.23)	231.6/234.6 (1.25 ± 0.55)	–	455.1/461.2 (39.41 ± 0.42)	456.2/462.2 (20.46 ± 0.65)	458.5/464.4 (40.13 ± 0.74)
MT-10/0	228.9/231.9 (14.41 ± 0.21)	231.7/234.7 (69.74 ± 0.54)	232.9/235.8 (15.85 ± 0.42)	455.2/461.3 (12.51 ± 0.64)	456.2/462.2 (0.06 ± 0.02)	458.7/464.5 (87.44 ± 0.64)
MT-10/10	228.9/231.9 (12.91 ± 0.57)	231.7/234.7 (70.45 ± 0.71)	232.9/235.9 (16.64 ± 0.13)	455.1/461.3 (13.17 ± 0.33)	456.2/462.2 (2.59 ± 0.2)	458.7/464.4 (84.24 ± 0.51)
MT-20/0	229.1/232.0 (10.47 ± 0.43)	231.8/234.8 (47.96 ± 0.55)	232.9/235.9 (41.57 ± 0.33)	455.3/461.3 (9.16 ± 0.55)	456.3/462.4 (0.04 ± 0.02)	458.7/464.5 (90.80 ± 0.55)
MT-20/10	229.1/232.1 (11.16 ± 0.51)	231.9/234.8 (42.49 ± 0.47)	232.9/236.0 (46.34 ± 0.29)	455.2/461.2 (10.8 ± 0.25)	456.3/462.4 (5.01 ± 0.17)	458.6/464.5 (84.19 ± 0.36)
MT-30/0	229.1/232.1 (0.27 ± 0.08)	231.8/234.7 (26.81 ± 0.73)	232.9/236.1 (72.92 ± 0.70)	455.2/461.2 (5.54 ± 0.36)	456.3/462.4 (0.04 ± 0.02)	458.7/464.5 (94.42 ± 0.38)
MT-30/10	229.2/232.1 (0.15 ± 0.02)	231.9/234.8 (26.45 ± 0.55)	232.9/236.0 (73.40 ± 0.53)	455.2/461.2 (1.78 ± 0.42)	456.3/462.4 (0.02 ± 0.01)	458.7/464.5 (98.20 ± 0.42)

As displayed in Fig. 4c, three oxygen components at binding energies of 530.3 eV (Ti–O–Ti), 532.1 eV (Ti–OH), and 531.2 eV (O–Mo) were observed in the O 1*s* spectra. Supplementary Table 3 presents a comprehensive summary of these results; specifically, the table presents information on the presence of molybdenum oxide and titanium oxide in the TM materials.

The change of different proportions of Mo elements with standard errors in MT materials are summarized in Supplementary Fig. 17a. The observed trend indicates a gradual increase in the proportion of Mo^5+^ and Mo^6+^ with an increase in laser power, whereas the content of Mo^4+^ initially exhibited a substantial decrease before stabilizing. This phenomenon can be attributed to the oxidation and modification of Mo^4+^ as a result of laser-induced pulses, leading to an increase in its valence state. The concomitant XPS data confirms that the modified Mo formed a more stable bond with oxygen, ultimately resulting in the formation of molybdenum oxide. Based on the results illustrated in Supplementary Fig. 17b, it can be inferred that the proportion of Ti–C bonds present in the MXene material decreased in response to an increase in laser power. In contrast, the proportion of Ti–O bonds showed a gradual increase under the same conditions. A slight change was noted in the composite material when the laser power remained unchanged but the pulse delay was adjusted. This is primarily attributed to the ionization of electrons and materials under the pulse delay. An increase in the pulse delay increased the valence states and the more uniform distribution of Mo and Ti in the composite material. This also confirms that the pulse delay excited more electrons, promoting complex chemical reactions including the redox reactions of Mo and Ti.

To further investigate the role of MT materials processed with varying laser parameters in electrochemistry, we conducted a study involving the testing of CV and galvanostatic charge-discharge (GCD) curves of electrodes fabricated from five groups of MT material. The areal capacitance of each electrode was then calculated based on the obtained curves. The mean value and standard error of the area ratio capacitance were used to generate a variation diagram (Supplementary Fig. 18). Additionally, we selected a material group with a capacitance value closest to the mean value for further analysis, presenting their respective CV and GCD curves (Supplementary Fig. 19). Among the tested materials, MT-20/10 exhibited the highest performance, which was consistent with previous characterization results. Subsequently, we employed this parameter for in-depth electrochemical research, utilizing a pulse laser energy of 20 mW and pulse delay of 10 ps.

### Electrochemical performance of the multitype MSCs

**Fig. 5:**
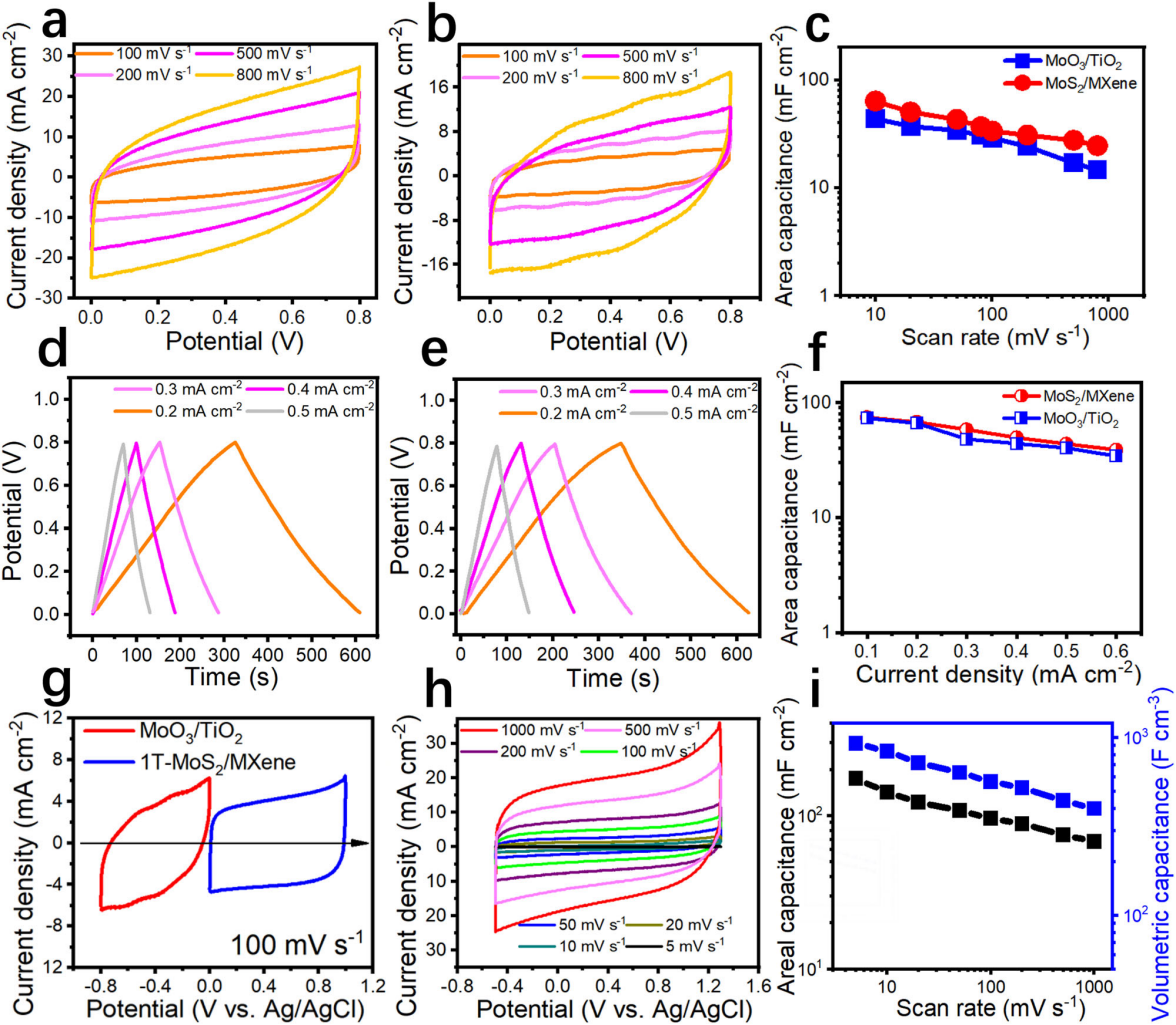
Electrochemical testing of different symmetric and asymmetric MSCs. **a**, **b** Cyclic voltammetry (CV) curves of the two symmetric MSCs under differing scan rates. **c** Comparison of the two MSCs’ electrochemical performance, as calculated from the CV curves. **d**, **e** GCD profiles of the two types of MSCs (MoO_3_/TiO_2_ and MXene/1T-MoS_2_) under differing current densities. **f** Comparison of the electrochemical performance of the two MSCs, as determined from the GCD profiles. **g** CV curves of the different electrodes at a scan rate of 100 mV s^−1^ measured in a three-electrode cell. **h** CV curves of 1T-MoS_2_/MXene//1T-MoS_2_-derived MoO_3_/MXene-derived TiO_2_ micro-supercapacitor at scan rates of 5–1000 mV s^−1^. **i** Summary of the electrochemical performance of the electrode materials under differing scan rates.

A set of anti-interference probe bench testing equipment, including a confocal observation system, probe bench electrochemical workstation, and anti-interference cover was developed to precisely evaluate the electrochemical performance of micro-supercapacitors (Supplementary Fig. 20). We tested the electrochemical properties of the various MSCs and compared their differences. We have chosen different symmetrical MSCs with a thickness of one micron and a size of 10 × 10 μm^2^ for performance comparison, in order to demonstrate the difference in capacitance characteristics of MSCs of different material types at such a small scale. Regarding the performance of the symmetric MSCs, Fig. 5a, b show the CV curves of the MSCs corresponding to the two-material systems (MXene/1T-MoS_2_ and MXene-derived TiO_2_ and 1T-MoS_2_-derived MoO_3_) in 1 M H_2_SO_4_ aqueous electrolyte. They all had a rectangular shape, indicating that the MSCs processed using this method have favorable capacitance features. As shown in Fig. 5c, the MXene/1T-MoS_2_ MSCs slightly outperformed the MXene-derived TiO_2_ /1T-MoS_2_-derived MoO_3_ MSCs. This is primarily attributed to the excellent conductivity and capacitance properties of two-dimensional materials; the conductivity of metal oxides was slightly inadequate in comparison. The galvanostatic charge-discharge (GCD) profiles of the two types of MSCs (Fig. 5d, e) are consistent with previous results. A performance comparison of the two MSCs with electrode materials was performed based on the GCD profiles (Fig. 5f). The electrochemical performance of 1T-MoS_2_/MXene and MXene-derived TiO_2_ /1T-MoS_2_-derived MoO_3_ in a three-electrode cell with 1 M H_2_SO_4_ as aqueous electrolyte were evaluated to explore the contribution of each component to the electrochemical performance of hybrid electrode materials in the symmetric MSCs (Fig. 5g). The measurement was performed in a three-electrode system, with 2 μm-thickness electrode films, Ag/AgCl electrode, and Pt sheet as working, reference, and counter electrodes, respectively. This indicates that the asymmetric device can stably operate at a cell voltage of up to 1.8 V. The mass ratio of the positive electrode and the negative electrode was optimized by balancing charges between the two electrodes (Q_+_ = Q_−_, where Q_+_ and Q_−_ are the amount of charge stored in the positive and negative electrodes, respectively). The CV curves of the electrodes were measured at scan rates ranging from 5 to 1000 mV s^−1^. Further, we computed the areal (189 mF cm^−2^) and volumetric capacitance (957 F cm^−3^) of electrode materials in the three-electrode system at different scan rates following the CV curves (Fig. 5h, i). Specific areal capacitance is calculated based on the addition of both positive and negative electrodes.

**Fig. 6:**
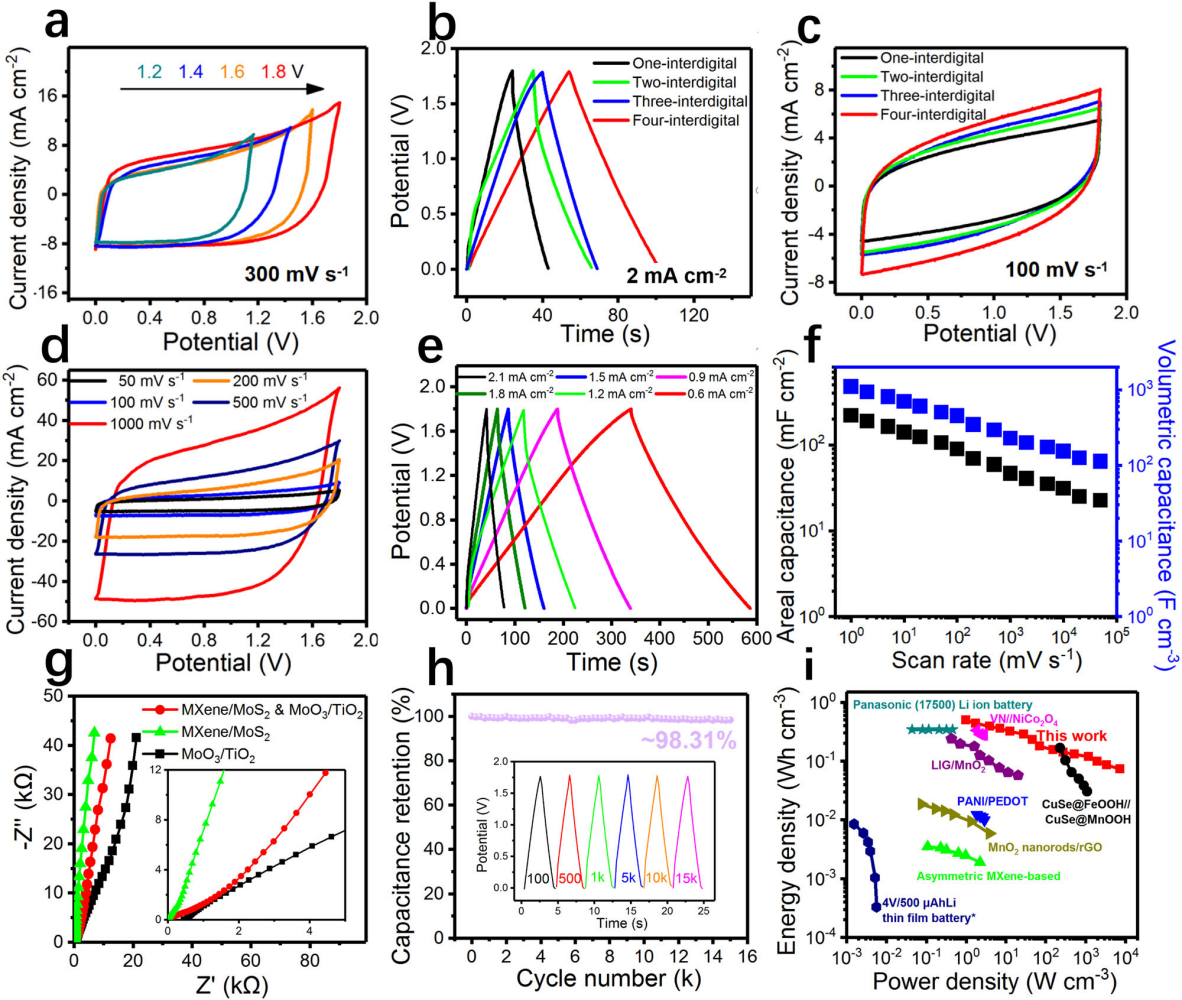
Electrochemical performance results and summary of asymmetric MSCs. **a** Cyclic voltammetry (CV) curves of the asymmetric MSCs in a gradually increasing voltage window. **b** GCD profiles of the asymmetric MSCs with different fingers in the 1.8-V voltage window. **c** CV curves of the asymmetric MSCs with different fingers at 100 mV s^−1^ scan rate. **d** CV curves of the asymmetric MSCs under differing scan rates. **e** GCD profiles of the asymmetric MSCs under differing current densities. **f** Summary of the electrochemical performance of the MSCs under differing scan rates. **g** Cycle life of the asymmetric MSCs (the independent GCD profiles in the illustration). **h** Ragone plot comparing the areal power density and energy density with other similar asymmetric MSCs. **i** Ragone plot comparing the volumetric power density and energy density with other MSCs and microscale energy storage devices.

The electrochemical storage mechanism of our hybrid electrode, electrical double-layer capacitance, and diffusion-limited capacitance contributions of the total capacitance was performed in Supplementary Figs. 21 and 22. This shows that the proportional capacitance is contributed by the capacitive process (electrical double-layer or outer surface) and the diffusion-limiting process (inner surface). Further, comprehensive electrochemical testing was performed on asymmetric MSCs with a thickness of 2 μm and a size of 10 × 10 μm^2^. As shown in Fig. 6a, favorable capacitance characteristics were retained even after expanding the voltage window from 1.2 to 1.8 V in 1 M H_2_SO_4_ aqueous electrolyte. Such a large voltage window promoted the energy density of asymmetric MSCs. The shapes, number of fingers, and microscopic size of the interdigitated MSCs were regulated through our approach. We first determined the impact of different shapes of MSCs (Supplementary Figs. 23 and 24). Interdigitated MSCs with various numbers of fingers were fabricated, and the effect of the number of fingers on electrochemical performance was investigated. Curves in GCD profiles generated under a similar current density of 1.8 V revealed favorable characteristics. The GCD profiles in Fig. 6b show that MSCs with more fingers outperform those with fewer fingers; this is associated with the area of the active interface site and shorter ion diffusion path. Multiple fingers can increase electrode-electrolyte contact and promote rapid ion transfer, thereby improving electrochemical performance. CV curves were measured based on the changes in scan rates. We noted a rectangular shape and similar curves (Fig. 6c). The specific capacitance results confirm that MSCs with more fingers have more favorable electrochemical performance. We assessed the electrochemical properties of MSCs with four fingers. Figure 6d, e shows the CV curves and GCD profiles corresponding to these MSCs under various current densities and scan rates, respectively. We established areal and volumetric capacitance of MXene-derived TiO_2_/1T-MoS_2_-derived MoO_3_//MXene/1T-MoS_2_ MSCs under changes in scan rate (220 mF cm^−2^ and 1101 F cm^−3^, respectively). Even under an extremely high scan rate of 500 V s^−1^, our MSCs exhibited ultrahigh electrochemical performance; in other words, our MSCs have favorable rate performance (Supplementary Fig. 25). An Agilent 4294 A impedance meter was used to test the capacitance contribution of substrate SiO_2_ in order to exclude potential interference. As illustrated in Supplementary Fig. 26, we computed the capacitance contribution value of 1 × 10^−6^, which is far less than the capacitance performance of the entire device. Nyquist plots of multitype MSCs (Supplementary Fig. 27) display slopes approaching 90° in a low-frequency range, indicating that the MSCs have excellent capacitance characteristics and can be used in various material systems. The MXene and 1T-MoS_2_ MSCs had the lowest equivalent series resistance of 0.55 mΩ cm^−2^. This value is extremely low in the field of MSCs, indicating extremely low internal resistance of the MSCs. No semicircles were detected, indicating a fairly low charge transfer resistance. Furthermore, the vertical straight line at the low frequency in the Nyquist plot confirms the rapid diffusion of ions to electrodes and the presence of electron transfer. We also tested the Bode phase angle plot (Supplementary Fig. 28). The characteristic frequency of our 1T-MoS_2_/MXene//1T-MoS_2_-derived MoO_3_/MXene-derived TiO_2_ supercapacitor at −45° was 54,687 Hz, corresponding to a time constant (τ0) of 0.018 ms, implying efficient ion transport/electron conductance. The slopes are extremely close to 90° (82.92°) at the frequency of 120 Hz. The MXene-derived TiO_2_/1T-MoS_2_-derived MoO_3_//MXene/1T-MoS_2_ MSCs exhibit a long cycle life and favorable cycling stability (Fig. 6g). After 15,000 cycles, the MSCs retained more than 98.8% of the initial capacitance. We extracted several GCD profiles from the loop for comparison, which were consistent under a voltage window of 1.8 V. CV curves of asymmetric MSCs under distinct bending states were generated (Supplementary Fig. 29). We further evaluated the environmental robustness of miniature supercapacitors. We aged the same micro-supercapacitor at room temperature for 45 days, and the CV curves before and after the aging are almost identical, exhibiting extremely high durability (Supplementary Fig. 30).

As displayed in the Supplementary Tables 4 and 5, we summarized the thickness, areal and volumetric power density, and energy density of similar ultra-thin micro-supercapacitors, and discovered the excellent performance of our micro-supercapacitors (55.97 mW cm^−2^ and 49.50 μW h cm^−2^), even better than devices thinner than ours (Fig. 6h)^36–43^. Figure 6i presents a Ragone plot that compares the energy and power density of our MSCs as well as other energy storage devices. The energy density of 0.495 W h cm^−3^ achieved is several orders of magnitude higher than that of other capacitors or batteries^22,44–47^. Additionally, our MSCs exhibited an excellent power density of 28,295 W cm^−3^ when the scan rate is high (Supplementary Fig. 25), which can be attributed to their facile electron and ion diffusion. Next, we tested the performance of the prepared MSCs in a solid PVA/H_2_SO_4_ gel electrolyte to confirm that they are compatible with other miniaturized devices. Supplementary Fig. 31 shows the CV curves and capacitance properties of the MSCs in the gel electrolyte, which are comparable to the electrochemical properties in the water-electrolyte. In the field of micro energy storage devices, achieving integrated manufacturing and high output voltage is crucial for practical application in various fields. To overcome this challenge, the confocal dispensing equipment with a 0.5-micron dispensing head is necessary for direct electrolyte coating and electrochemical testing of individual MSCs, while simultaneously avoiding diffusions and leakage currents. This equipment is depicted in Supplementary Fig. 32. However, in the case of large-scale integrated series manufacturing of multiple MSCs, a lithograph-assisted approach is required. This method involves the exposure of the MSCs in series requiring electrolyte through a mask plate design. Subsequently, the high-precision dispensing machine is employed for drip-coating to ensure that each individual MSC is in a separate electrolyte environment, thus minimizing the likelihood of short circuits. As shown in Supplementary Fig. 33, the connectivity of thirty MSCs in a series configuration is facilitated through the use of lithography and dispensing techniques. This approach allows for the integration of MSCs with a considerably enhanced voltage window of 52 V (Supplementary Fig. 34). The leakage current of our MSCs can be measured by applying a rated DC voltage to a MSC and measuring the current required to maintain that voltage. And we can also obtain the self-discharge curve immediately after recharging MSC to *V*_max_. Supplementary Fig. 35a shows that the leakage current of our MSC is only 3.4 nA after 16 h, which is lower than the commercial and other MSC. Due to its low leakage current, the MSC can be integrated with energy harvesters to create efficient self-powered systems. Supplementary Fig. 35b shows that the self-discharge time of our MSCs is 10 h, which maintain 10 h from 1.8 V to 0.95 V. In summary, our MSCs have a high potential for use in microscale energy storage devices. Simultaneously, the scope of the application of the aforementioned technology is being extended to other micro devices. As depicted in Supplementary Fig. 36, an array of electrical and sensor components has been produced utilizing TSSF and incorporated with MSC, demonstrating successful applications in gas sensing detection (Supplementary Fig. 37).

## Discussion

This work realized the ultrafast maskless fabrication of multipatterned high-performance multitype MSCs and photochemical regulation of the synthesized composite materials by leveraging the special benefits of TSSF. The proposed methodology is highly versatile and can be employed for various material systems, including the development of asymmetric electrode structures from laser-induced materials. The MXene-derived TiO_2_/1T-MoS_2_-derived MoO_3_//MXene/1T-MoS_2_ MSCs had ultrahigh specific capacitance (220 mF cm^−2^ and 1101 F cm^−3^), cycling stability (98.3% capacitance retention after 15,000 cycles), energy density (0.495 Wh cm^−3^ and 49.50 μW h cm^−2^), and power density (28 kW cm^−3^ and 55.97 mW cm^−2^). The new micromachining technology is poised to significantly advance the industrial and integrated preparation of miniature functional devices, as well as promote the utilization of MSCs in the system-on-chip field. The prepared flexible and stretchable planar MSCs have the potential to be incorporated into a diverse range of applications, including artificial skin, wearable electronics, and even brain-computer interfaces.

## Methods

### MXene/1T-MoS_2_ hybrid thin film preparation

The single-layer MXene dispersion (Ti_3_C_2_, 200~500 nm, 2.5 mg mL^−1^) was derived from Nanjing XF Nano Material Tech Co., Ltd. The Ti_3_AlC_2_ MAX phase powder was sieved (particle size < 40 µm). The single-layer 1T-MoS_2_ nanosheet dispersion (200~300 nm, 2.5 mg mL^−1^) was purchased from Nanjing/Jiangsu XFNANO Materials Tech Co., Ltd. The two solutions separately underwent exfoliation for 5 h. Subsequently, the two diluted mixed solutions were mixed, subjected to sonication for 2 h, and stirred for 1 h. The prepared mixed solution was vacuum-filtered using a fiber filter. After 6 h of filtration, a layer of mixed film was produced, before vacuum-drying. Nitrocellulose membrane for vacuum filtration was purchased from Merck Millipore Ltd. The acetone was obtained from Sigma Aldrich and used to dissolve the cellulose film when transferring the mixed film. In a simple vacuum filtration process, MXene and monolayer MoS_2_ nanosheets were filtered through a membrane with an aperture of 25 nm to form a stacked MXene/metal 1T-MoS_2_ membrane. The thickness of this re-stacked film was adjustable based on the volume of the filtered solution. In our test, the thickness of the composite film prepared was 1μm.

### The temporally and spatially shaped femtosecond laser

A stable titanium sapphire laser regeneration amplification system was used to transmit a Gaussian beam with a central wavelength of 800 nm and a pulse duration of 35 fs. The maximum threshold for generating laser energy is 2 W. Holoeye Pluto (spatial light modulator) can receive the phase difference distribution of the load and reflect the beam away. The pulse laser energy and the pulse delay of the femtosecond laser were 20 mW and 10 ps. The designed electrode shape determines the intensity distribution by locating a 256 × 256-pixel region to a black 1080 × 1920 background image. An improved Gaussian algorithm was used to optimize the algorithm by increasing the number of iterations and a function to optimize the distance between the beam spots. Therefore, different expected light fields could be obtained. Thereafter, the gray phase hologram was loaded onto SLM to transform the light field of any geometry. Michelson interferometer was used to obtain subpulses with different pulse delays (from a few hundred femtoseconds to the picoseconds). The shaped beam was focused by an Olympus objective lens (20×, NA = 0.45). The sample was horizontally placed on the six-axes translation stage (M840.5DG, PI, Inc.).

### Characterization of laser-induced MXene/1T-MoS_2_

The morphology and microstructures were characterized by scanning electron microscope (SEM) using SU8220 (Hitachi, Japan) by a Hitachi SEM in Tsinghua university. X-ray photoelectron spectroscopy (XPS) analysis was performed using an ESCALAB 250Xi spectrometer with a monochromatic Al Kα source (7.5 µm beam spot). Raman spectra were acquired using a Via-reflex spectroscopy with the excitation laser line at 532 nm. The XRD patterns were performed on a D8 Advance (Bruker) with CuKa radiation. An Olympus metallographic microscope can take optical microscopy images. The Confocal Laser Scanning Microscopy used an MPLAPONLEXT x20 lens. A Renishaw inVia Reflex spectrometer with a laser wavelength of 532 nm was used to investigate Raman spectroscopy.

### Electrochemical characterization of the result of micro-supercapacitors

Additional analysis was performed to establish current density using the software package NanoScope Analysis. Electrochemical testing was conducted on a CHI760E electrochemical workstation connected to a Probe Station with polyamide-coated platinum probes (tip diameter, approximately 1 µm) as the current collectors. All our electrochemical tests are performed in atmospheric environments and at room temperature of 28 degrees Celsius. To ensure a stable electrochemistry environment, the open-circuit potential (Eocp) measurements were tested for one hour until we obtained a fluctuation of less than 10 mV 10 min before every electrochemistry measurement. During the electrochemical test, we set the potential cut offs to 1.8 V and the peak current to 0.1 mA. The electrochemical performance of the MSCs was measured in a two-electrode system and analyzed based on cyclic voltammetry (CV), galvanostatic charge/discharge (GCD), and electrochemical impedance spectra (EIS). The areal capacitance (mF cm^−2^) per electrode was derived from the CV and GCD tests using Eqs. (1) and (2), respectively, as follows:

1 $$C=\frac{1}{\vartheta \times V}\int \begin{array}{c}Vf\\ Vi\end{array}I(V)dV$$

where I, ϑ, and V represent the current applied, scanning rate, and voltage (Vf and Vi are the final voltage and initial voltage).

2 $$C=\frac{I}{(-dV/dt)}$$

where I is the discharge current, and *dV*/*dt* is the slope of the discharge curve. Cycling stability measurements were performed by repeating constant current charge-discharge at 1 mA for 15,000 cycles. The energy densities (mWh cm^−2^) of the supercapacitors were calculated according to the following equations:

3 $${E}_{{{{{\rm{cell}}}}}}={C}_{{{{{\rm{cell}}}}}}\varDelta {E}^{2}/(2\times 3600)$$

where ∆E represents the operating voltage window. Therefore, the power density (μW h cm^−2^) of the obtained supercapacitor was obtained as follows:

4 $${P}_{{{{{\rm{cell}}}}}}={E}_{{{{{\rm{cell}}}}}}\times 3600/t$$

where *t* represents the discharge time (*t* = ∆*V*/*ϑ*).

The fact that the size of our capacitor is tens of microns warrants attention; thus, extreme care should be paid to the capacitance computation. The above formula was used to simultaneously compute the capacitance of electrodes and the tungsten probe in the same electrolyte, which were together measured, respectively. The result corresponds to the true capacitance value *C*_cell_= *C*_electrodes_ − *C*_tungsten probe_; this eliminates interference from the tungsten probe.

### Reporting summary

Further information on research design is available in the Nature Portfolio Reporting Summary linked to this article.

## Supplementary information

Supplementary Information

Description of Additional Supplementary Files

Supplementary Movie 1

Supplementary Movie 2

Reporting Summary

## Data Availability

All relevant data that support the plots within this article and other findings of this study are available from the corresponding authors upon request. Source data are provided with this paper.

## Source data

Source Data
