# Supplementary material for: Laser maskless fast patterning for multitype microsupercapacitors
Source: Nat Commun. 2023 Jul 5;14:3967. doi: 10.1038/s41467-023-39760-3 (PMC10322851; doi:10.1038/s41467-023-39760-3)
Supplement: Supplementary file 1 — Supplementary Information [file 41467_2023_39760_MOESM1_ESM.pdf]

Supplementary Information

Laser Maskless Fast Patterning for Multitype Microsupercapacitors

Yongjiu Yuan[1,2,3,4], Xin Li[1,2,3]✉, Lan Jiang[1,2,3]✉, Misheng Liang[5], Xueqiang Zhang[1,2,3], Shouyu Wu[1,2,3], Junrui Wu[1,2,3], Mengyao Tian[1,2,3], Yang Zhao[6] and Liangti Qu[7]

[1]Laser Micro/Nano-Fabrication Laboratory, School of Mechanical Engineering, Beijing Institute of Technology, Beijing, P. R. China.

[2]Yangtze Delta Region Academy of Beijing Institute of Technology, Jiaxing, P. R. China.

[3]Beijing Institute of Technology Chongqing Innovation Center, Chongqing, P. R. China.

[4]Department of Mechanical Engineering, City University of Hong Kong, Hong Kong, P.R. China.

[5]School of Instrument Science and Opto-Electronics Engineering, Beijing Information Science and Technology University, Beijing, P. R. China.

[6]Key Laboratory of Cluster Science Ministry of Education of China, School of Chemistry and Chemical Engineering, Beijing Institute of Technology, Beijing, P. R. China.

[7]MOE Key Laboratory of Bioorganic Phosphorus Chemistry & Chemical Biology, Department of Chemistry, Tsinghua University, Beijing, P. R. China.

✉e-mail: lixin02@bit.edu.cn; jianglan@bit.edu.cn

Supplementary Figure

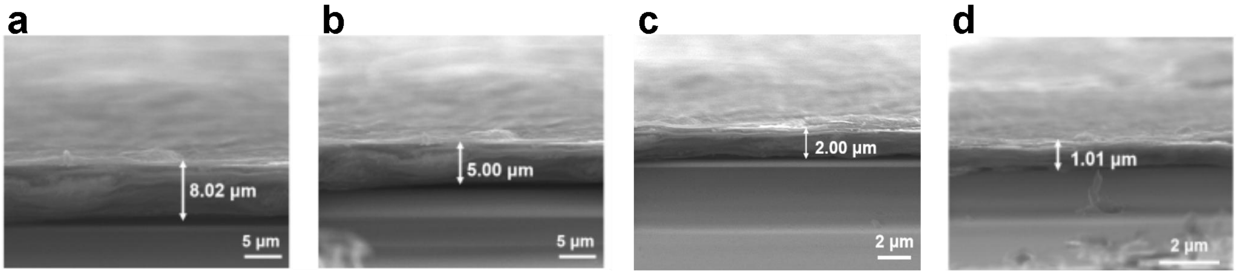

Supplementary Figure 1: a-d 1T-MoS_2/MXene hybrid films of different thicknesses were obtained by controlling the amount of mixed two-dimensional material solution by vacuum filtration. The SEM image of the prepared films clearly shows that the thickness of different films is 1, 2, 5, 8 microns.

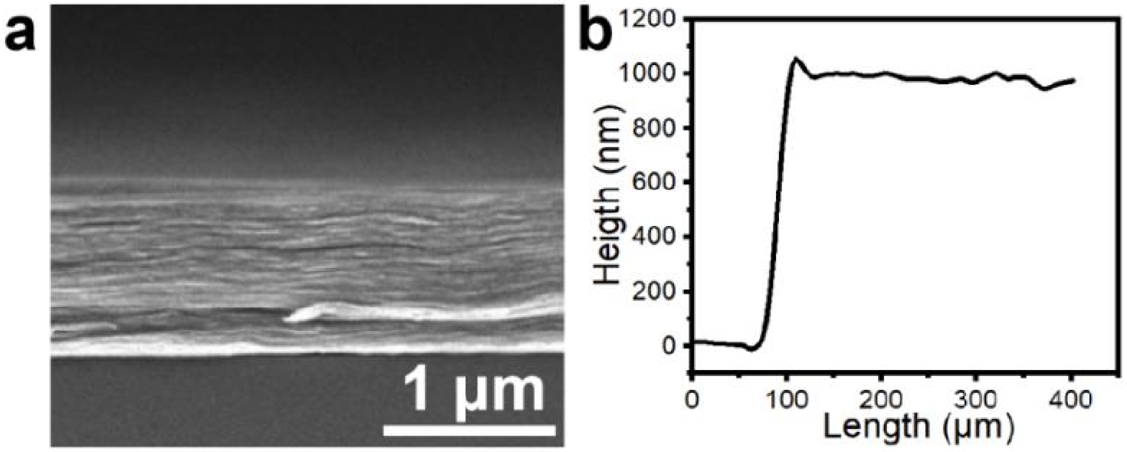

Supplementary Figure 2: a Scanning electron microscopy (SEM). **b** the atomic force microscopy (AFM) images of the film surface were obtained.

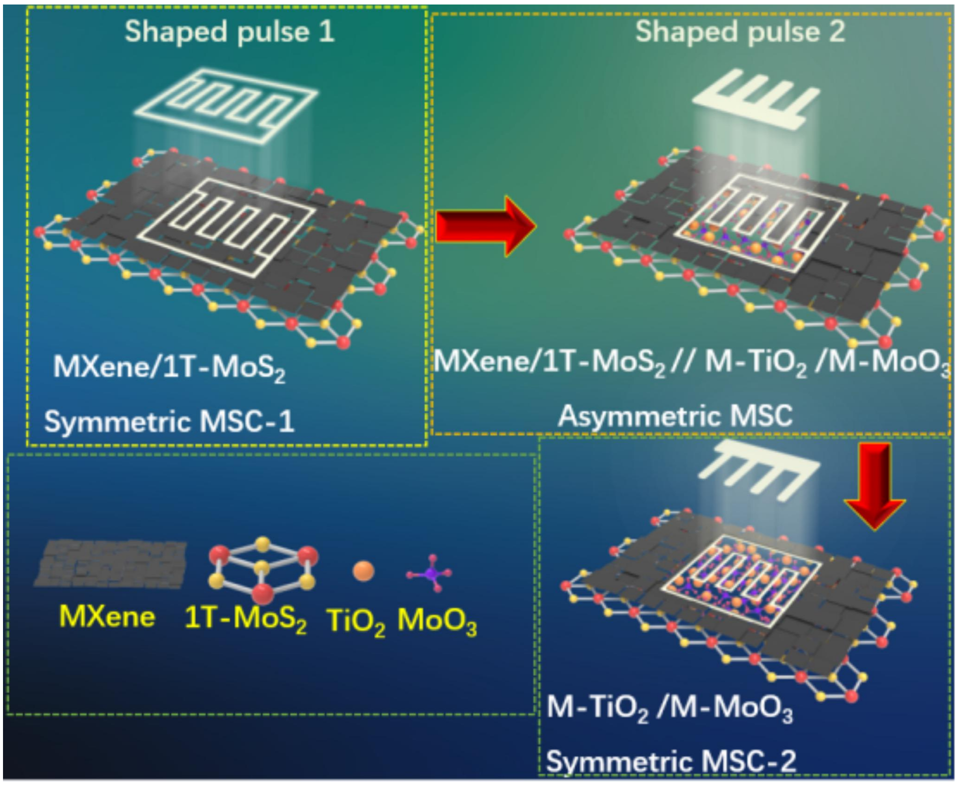

Supplementary Figure 3: The Schematic of the laser-induced synthesis of materials for different MSCs. The original material (1T-MoS_2/MXene) was irradiated with different-shaped pulse lasers, and shaped light field 1 was used to remove the 1T-MoS_2/MXene. Further, light fields 2 and 3 were shaped to modify the material in order to obtain laser-induced MXene-derived TiO_2/1T-MoS_2-derived MoO_3. Consequently, the whole process realizes two processes laser-induced material synthesis and laser removal.

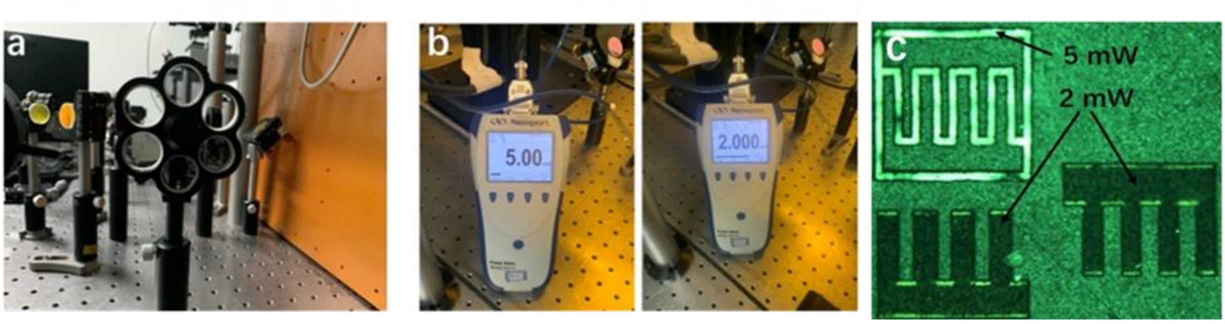

Supplementary Figure 4: Throughout our experiment, the spatially shaped femtosecond laser ablated the material of two different processing, respectively for ablative modification and ablative removal. Therefore, we adjusted the laser energy to make it not attenuated in the processing, so that the material can be completely removed. In our experimental exploration, it is found that the two-dimensional material film can be completely removed when the focusing power above 5 mW is used for processing. When we attenuated the laser energy with a laser attenuator (as shown in Figure a), it was found that the laser power could be adjusted to about 2 mW, and the power could be accurately measured with a power meter (Figure b). As shown in Figure c, the area has obviously been completely removed by large laser energy (5 mW), and we found no direct transmission of light through this processing area under the processing area with the laser power of 2 mW, indicating that the material has been modified rather than completely removed.

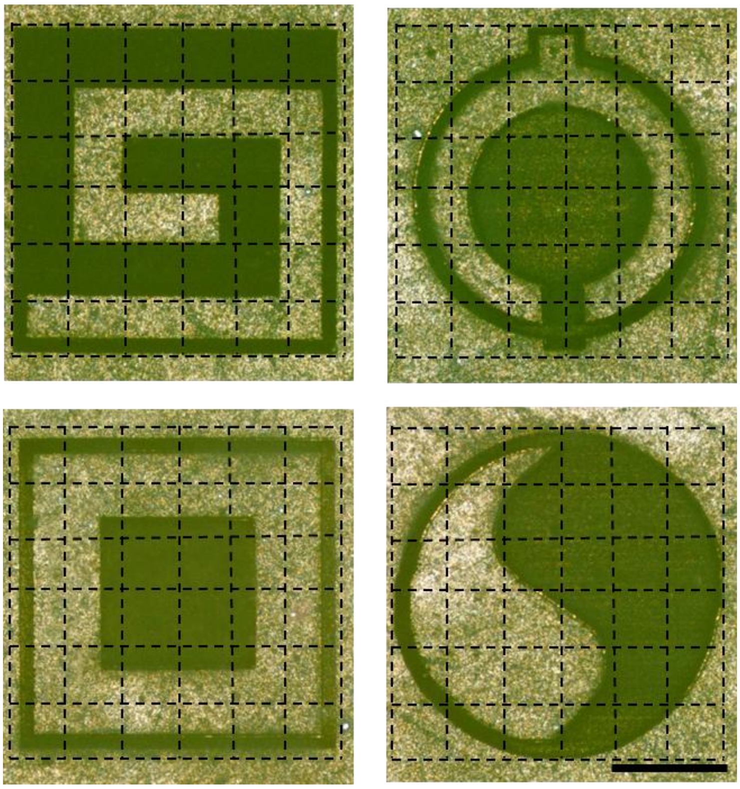

Supplementary Figure 5: The optical micrographs of asymmetric MSCs with a variety of personalized patterns. We can use this technology to achieve large-scale processing, and use the reshaped laser light field to complete the stitching in a short time (Each small square represents a sub-pulse shaped light field, which can be spliced into a large-scale pattern by using different pulse light fields). This proves that our technology can achieve pattern processing from small size to large size (scale bar, 500 μm).

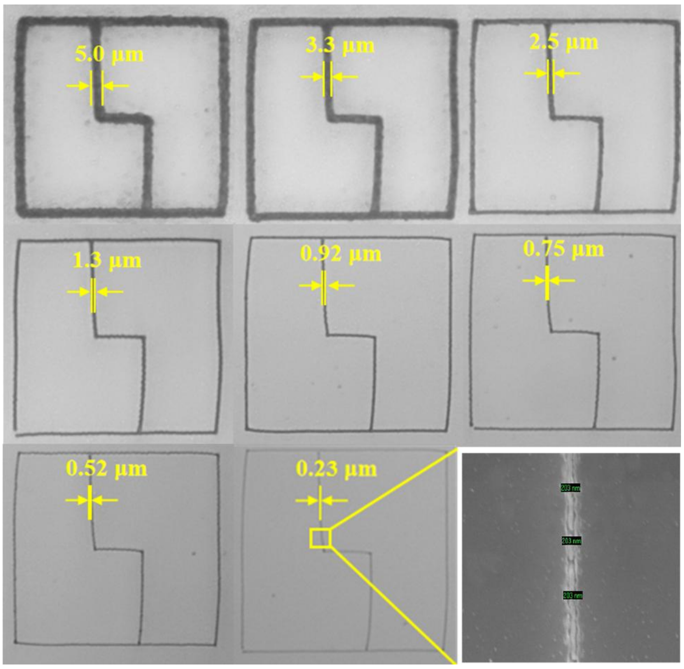

Supplementary Figure 6: MSCs patterns with different line widths. We can design patterned MSCs with different linewidths by adjusting the patterns and parameters of the light field using the shaped laser. These line widths can be adjusted from micron to nanometer scale.

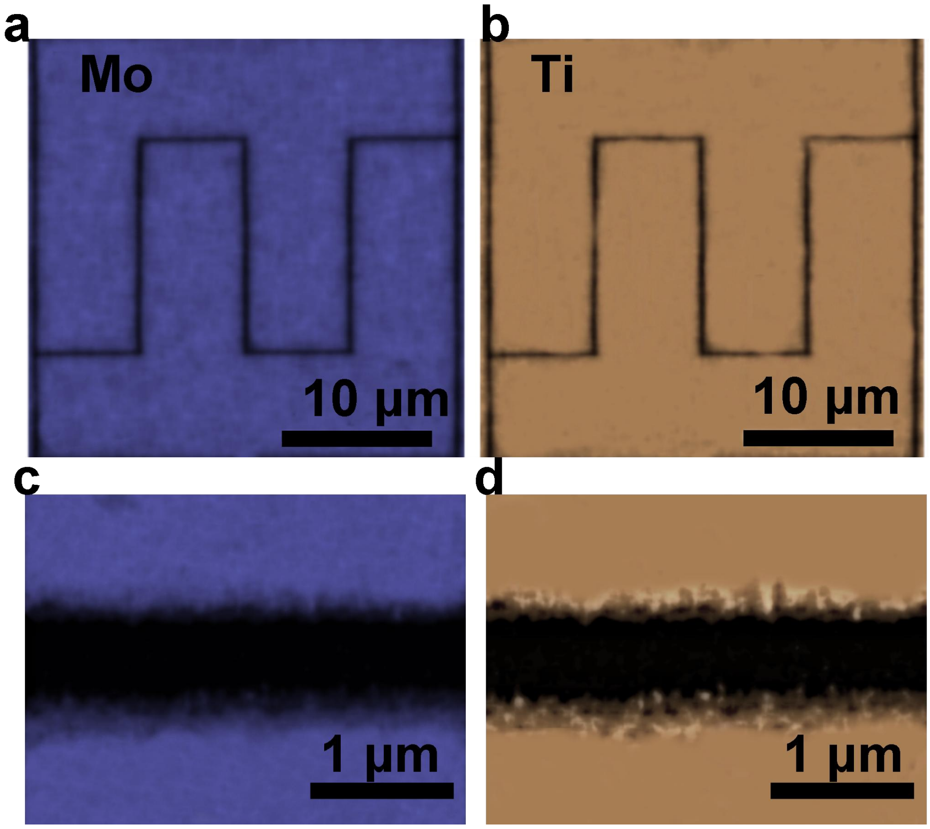

Supplementary Figure 7: The element mapping of our MSCs. **a** and **c**, Element mapping (Mo) of the patterned MSCs and the high-resolution lines. **b** and **d**, Element mapping (Ti) of the another patterned MSCs and the high-resolution lines.

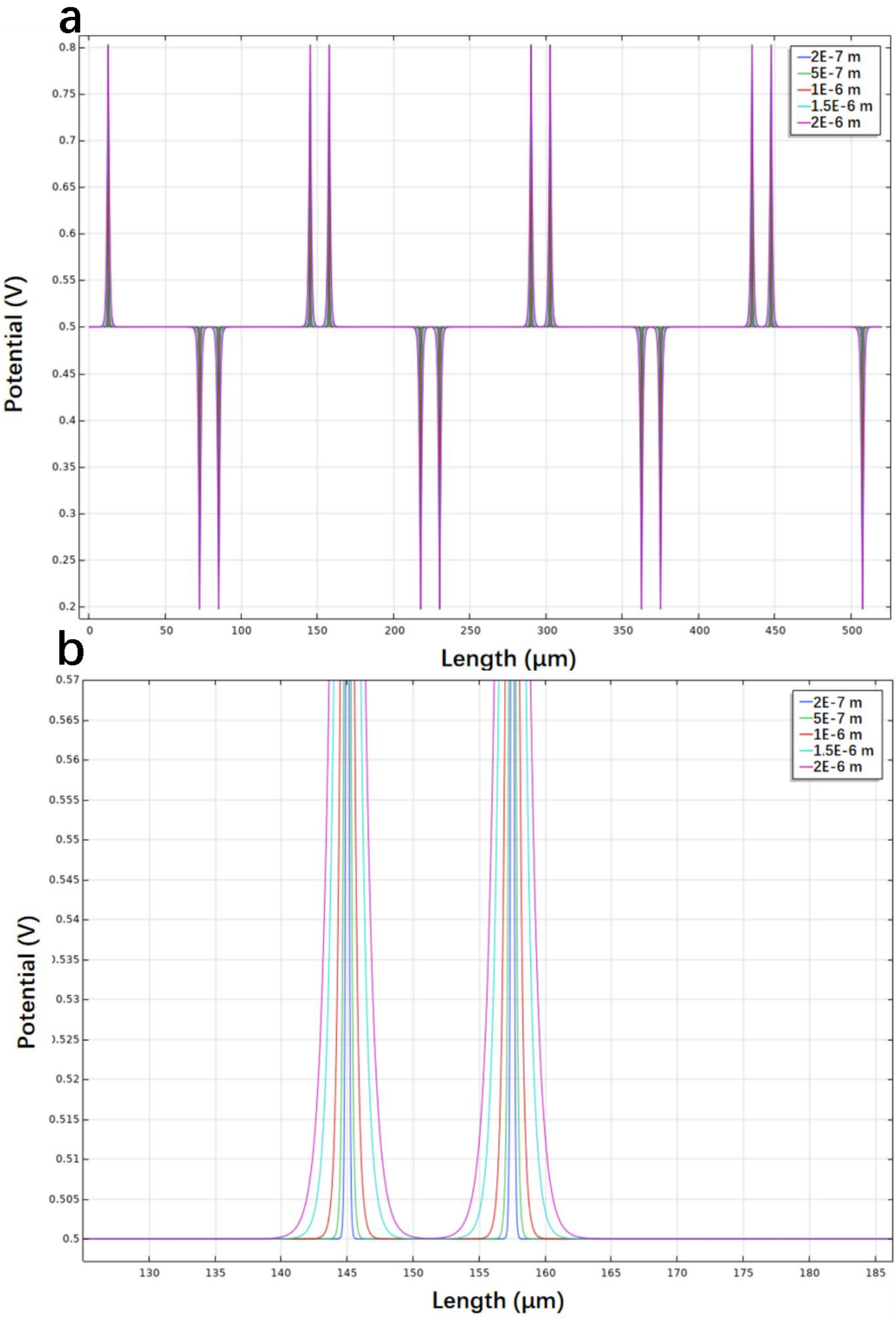

Supplementary Figure 8: a Potential distribution curve of the micro supercapacitor at the narrow gap. **b** Partial magnification of the potential distribution.

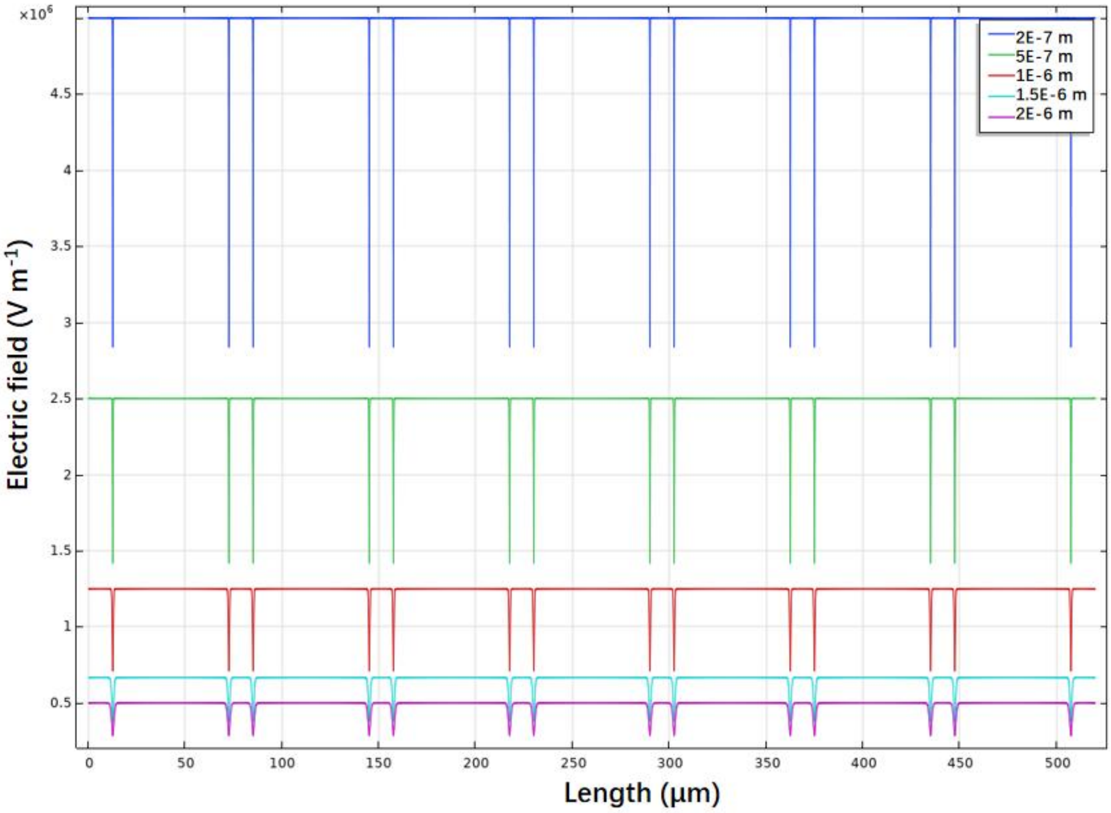

Supplementary Figure 9: Electric field distribution curves of the micro-supercapacitor at the narrow gap.

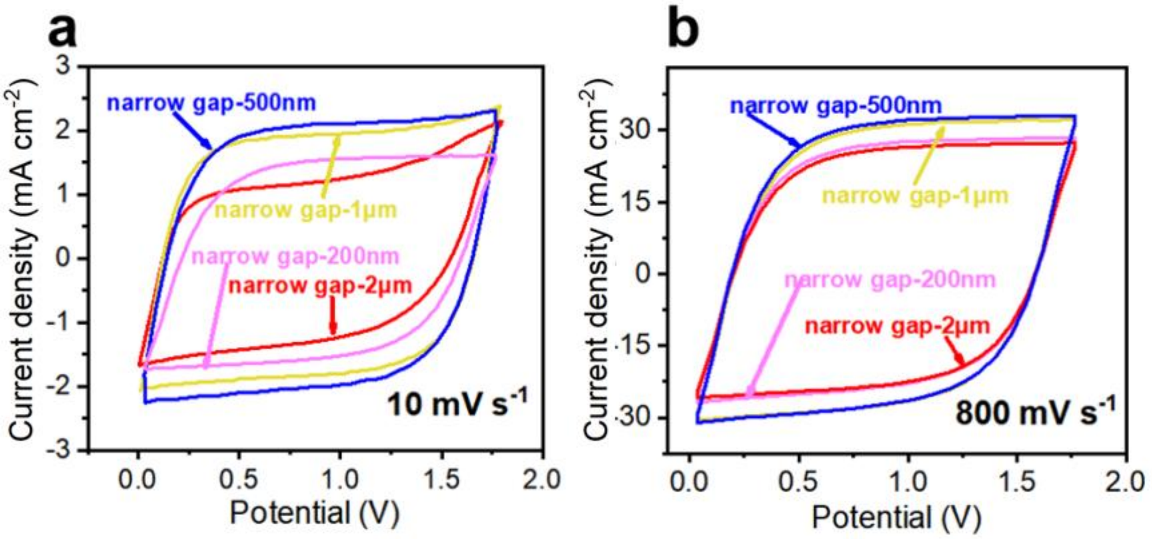

Supplementary Figure 10: The comparison of electrochemical performances (CV characterizations at (**a**) 10 and (**b**) 800 mV s^{-1}) of the micro-supercapacitors with different narrow gaps.

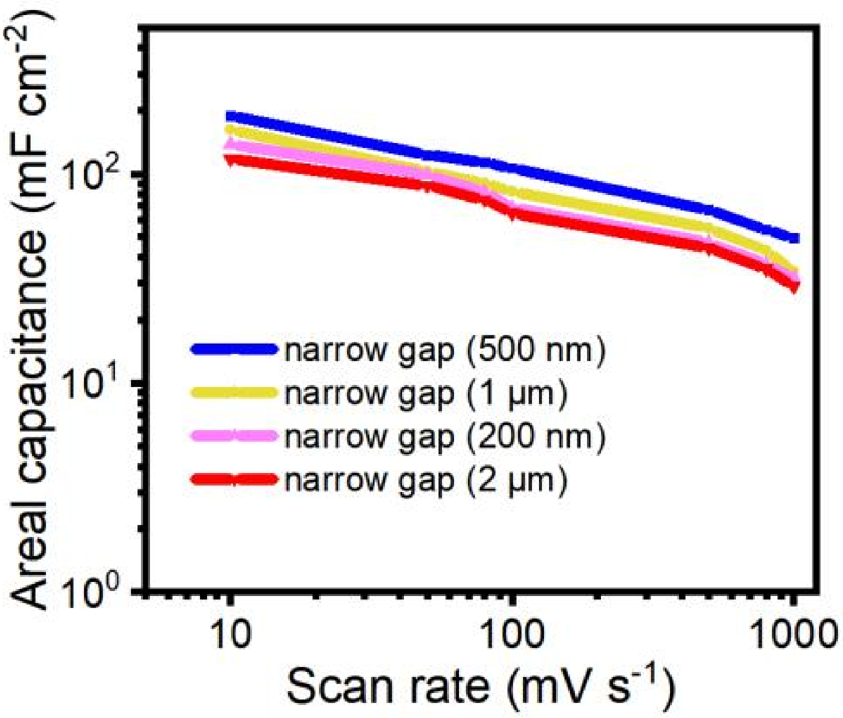

Supplementary Figure 11: The areal capacitance of the micro-supercapacitors with different narrow gaps as a function of scan rates.

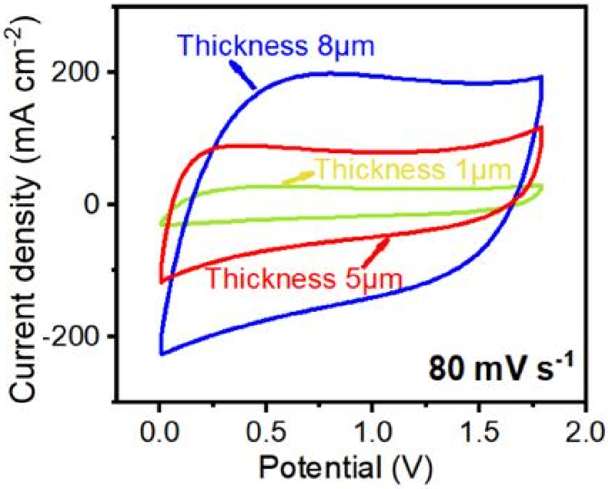

Supplementary Figure 12: The comparison of CV characterizations at 80 mV s^{-1} of the micro-supercapacitors with different thicknesses.

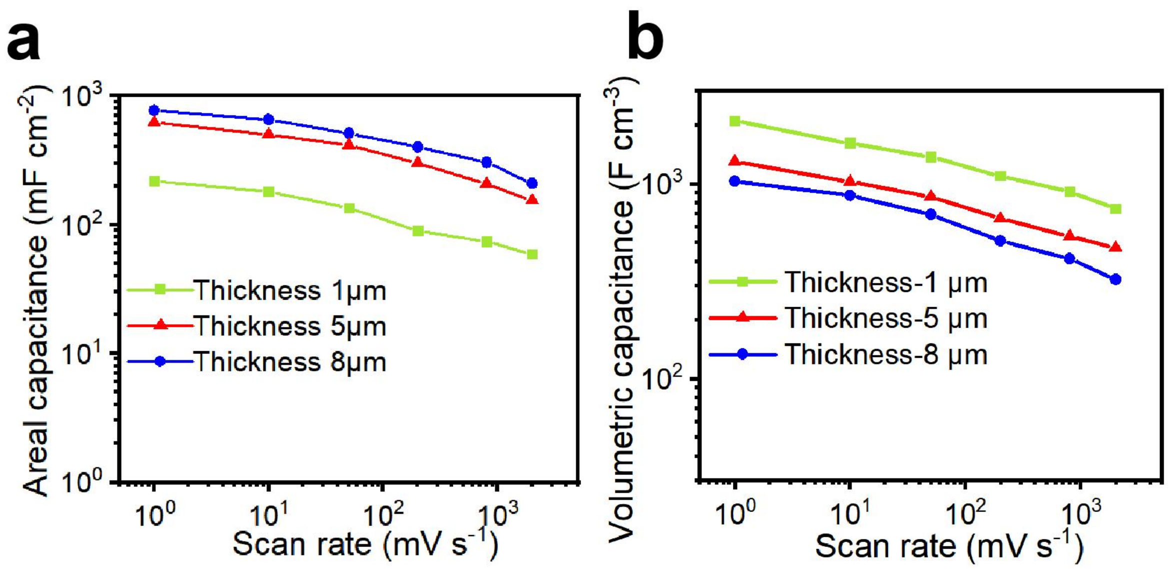

Supplementary Figure 13: Areal (**a**) and volumetric (**b**) capacitance of micro-supercapacitors with different thicknesses as functions of the scan rate.

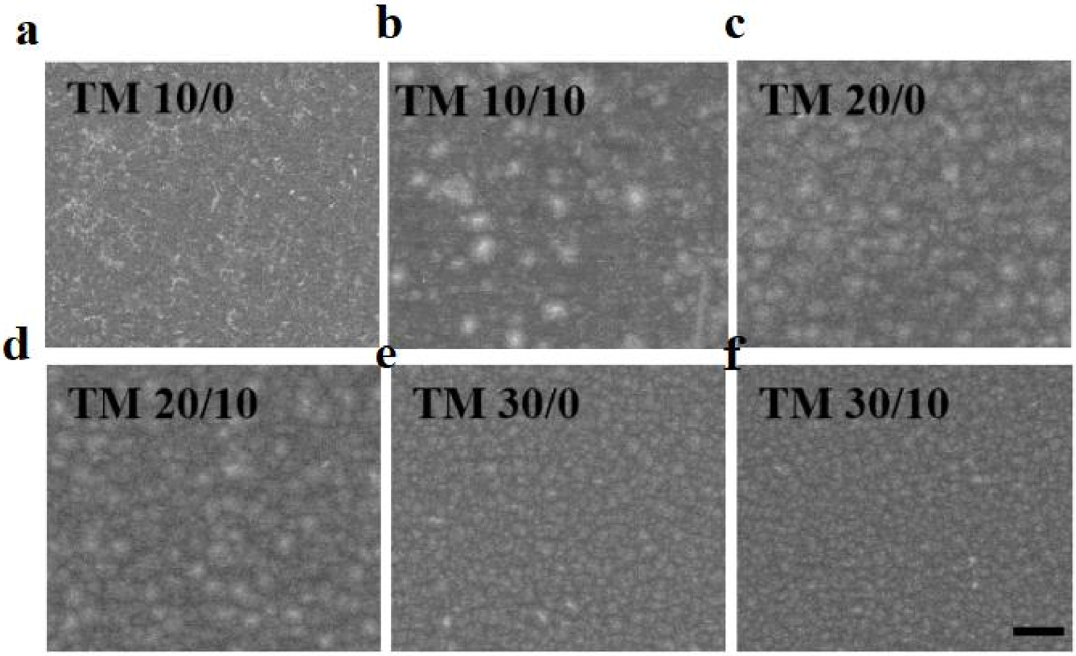

Supplementary Figure 14: a-f The SEM images of 1T-MoS_2/MXene composite films processed by different shaped laser parameters (scale bar, 1 μm). With the increase of laser energy, it can be found that the particle size of composite surface decreases gradually, which is related to the cumulative ablation of laser. By adjusting the delay of the two sub-pulses, it can be found that the morphologies of the modified materials are slightly different.

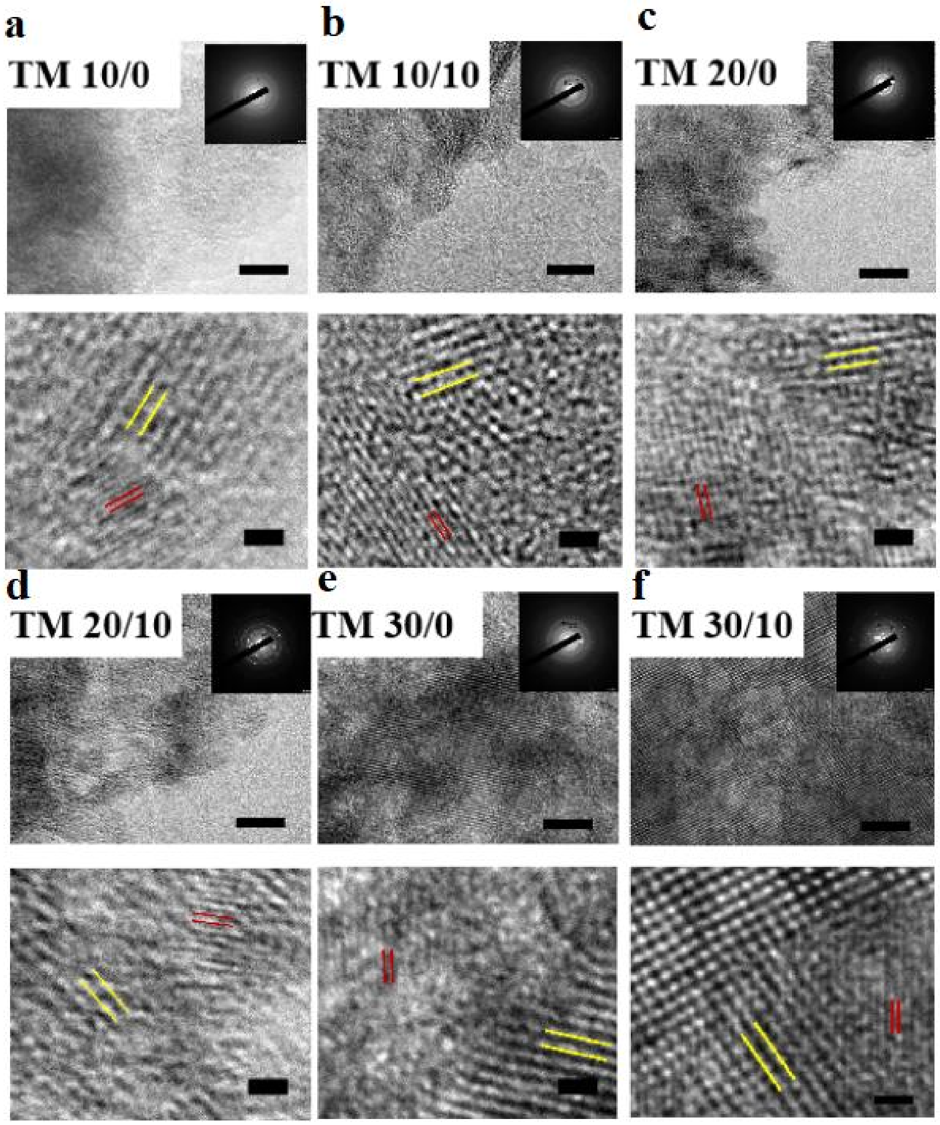

Supplementary Figure 15: TEM and high-resolution TEM (**a-f**) of laser-induced materials with different laser parameters showed that MoO_3 and TiO_2 existed in the laser-induced composites, which also proved that the oxidation of metal oxides was realized in our processing process.

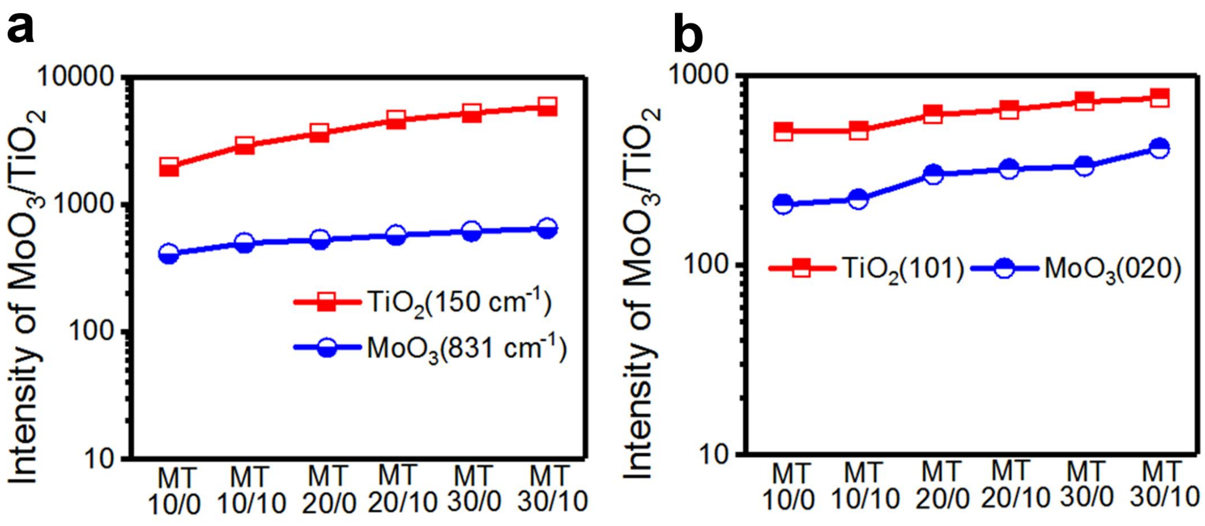

Supplementary Figure 16: a The intensity of laser-induced MoO_3/TiO_2 under different laser parameters according to Raman spectra. **b** The intensity of laser-induced MoO_3/TiO_2 under different laser parameters according to XRD spectra.

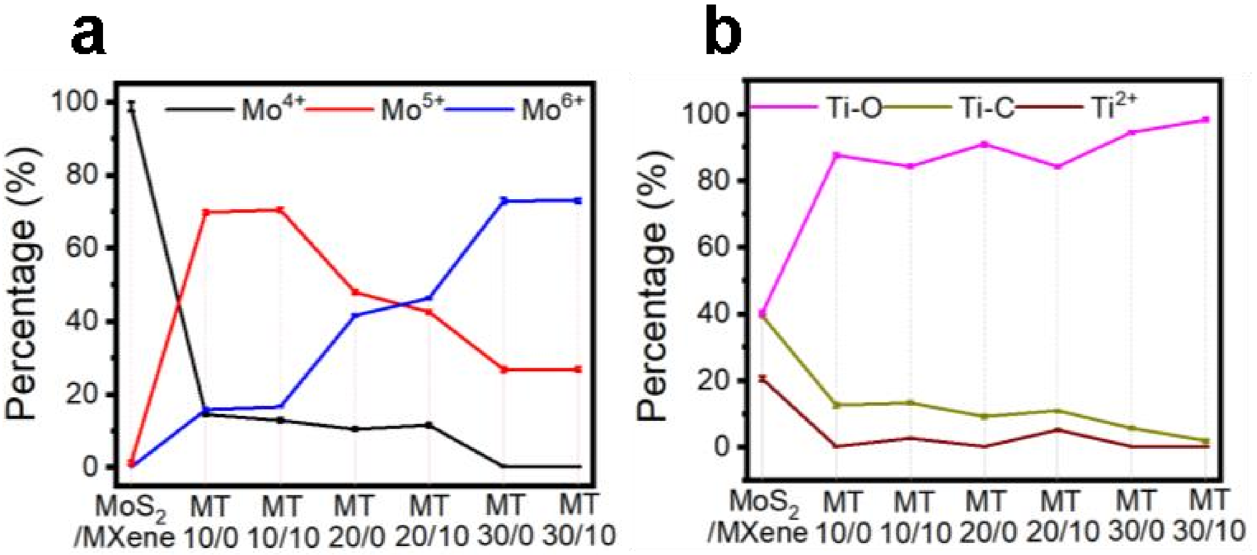

Supplementary Figure 17: The change of different proportions of Mo (**a**) and Ti (**b**) elements with standard errors in MT materials. Reported values correspond to the average of triplicate size measurements. Error bars represent the standard error of the mean.

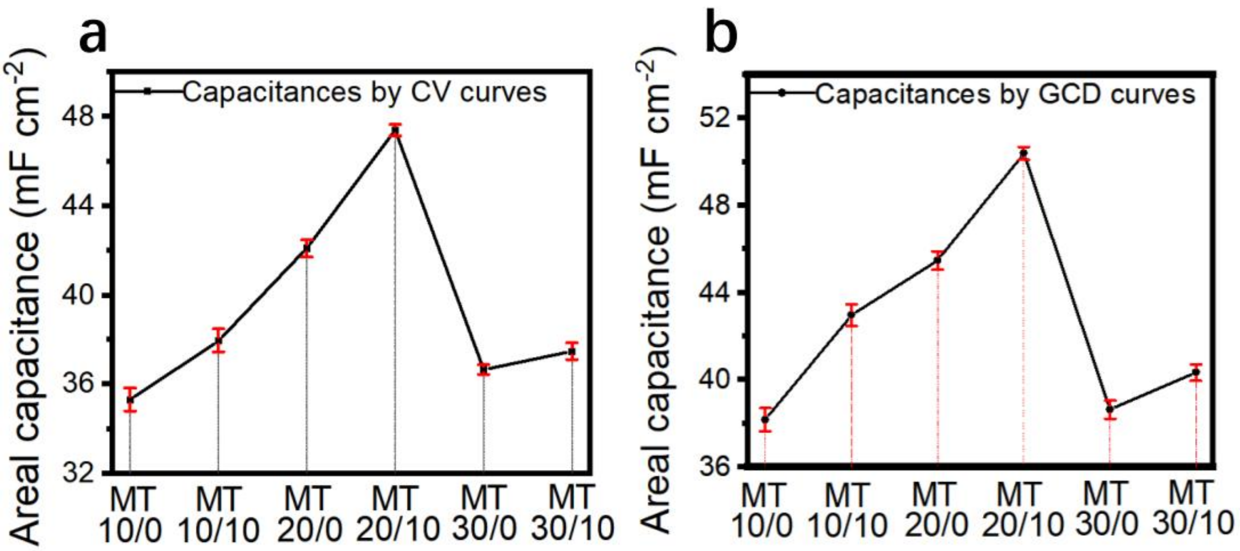

Supplementary Figure 18: The changes of the areal capacitance in different composite material according to CV (**a**) and GCD (**b**) curves. Reported values correspond to the average of triplicate size measurements. Error bars represent the standard error of the mean.

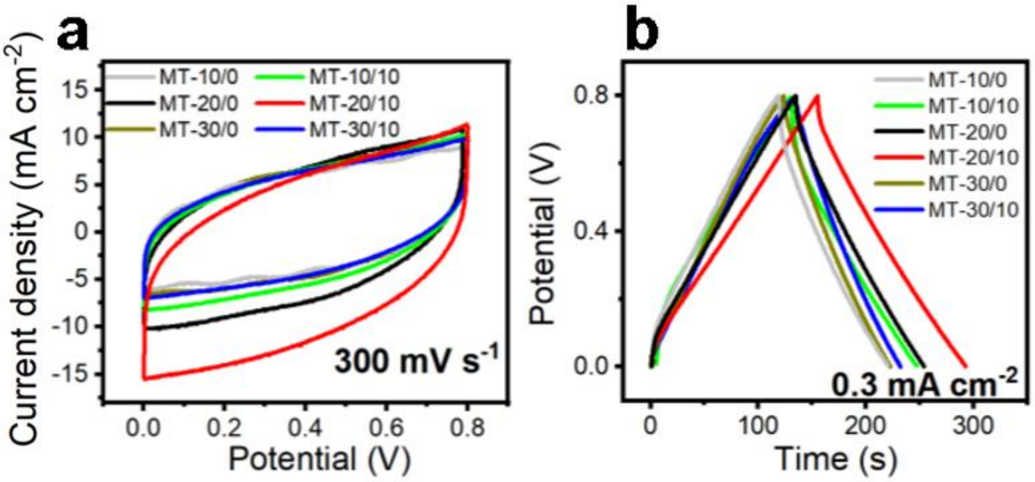

Supplementary Figure 19: a The CV curves of different MT materials at the scan rate of 300 mV s^{-1} and **b** GCD curves of different MT materials at the current density of 0.3 mA cm^{-2}.

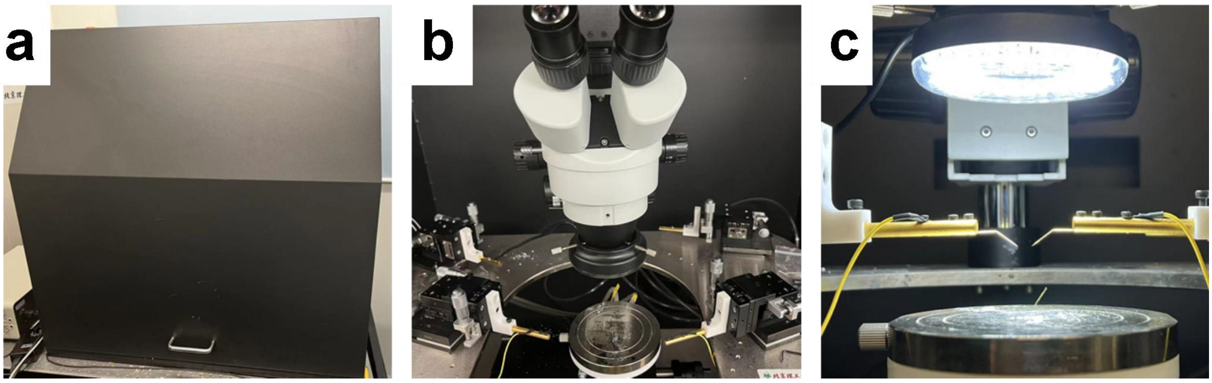

Supplementary Figure 20: Probe Station with polyamide-coated platinum probes. **a** Shielding cover of probe station, **b** optical microscope of the probe station, **c** polyamide-coated platinum probes (tip diameter, approximately 1 μm).

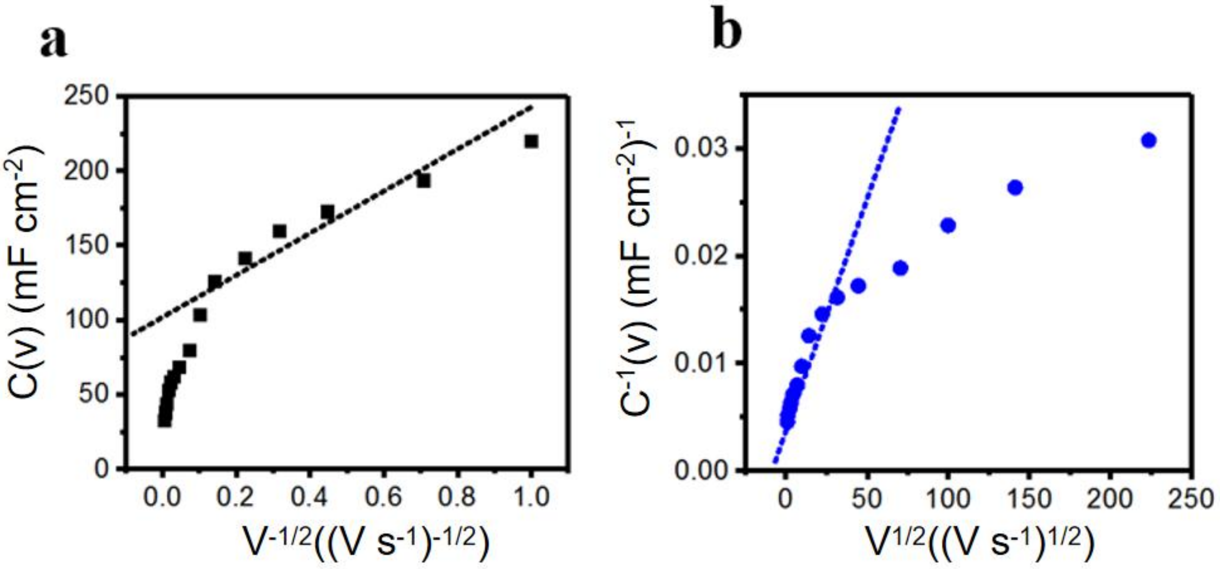

Supplementary Figure 21: **a** and **b** The total voltametric charge (q_T) is obtained from the contribution of the outer surface of the electrode (q_o) and the inner bulk of electrode (q_i): $q_T = q_o + qi$. As $v \rightarrow \infty$, $q(v) = q_o + C_1 v^{-1/2}$, where q (v) is the measured voltametric charge and C_1 is a numerical constant; as v goes to $0, 1/q(v) = 1/q_T + C_2 v^{1/2}$, where C_2 is a numerical constant. We did the research on our MXene/graphene as described above. The capacitance is directly proportional to the charge: C=q/V, where V is the potential window used. The diffusion-limited capacitance C_D then can be calculated by subtracting the value of the electrical double layer capacitance from the value of total capacitance C_T from the figure. We can get C_D Thus, we can obtain the proportions of C_{DL} and C_D in the total C_T, indicating that MXene and Graphene alone provide their respective capacitance contributions.

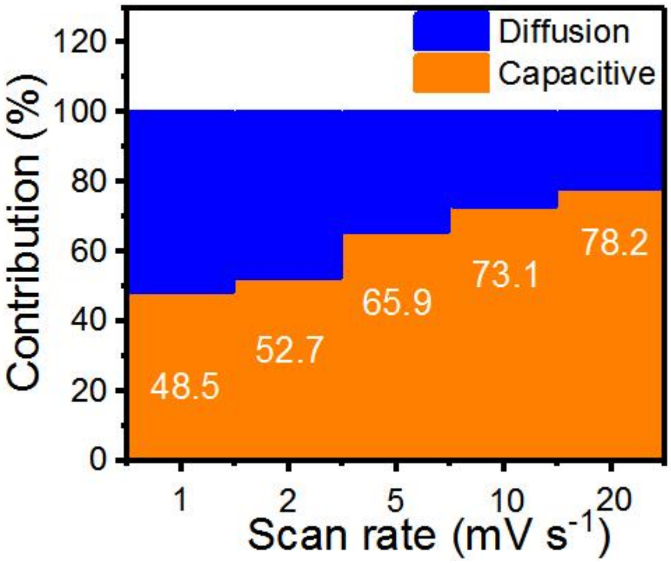

Supplementary Figure 22: The ratio of the diffusion-limited and capacitive capacitance at different scan rates. It can be obtained that the proportion of capacitance contributed by the capacitive capacitance in the hybrid electrode with different scan rates (1, 2, 5, 10 and 20 mV s^{-1}) is 48.5%, 52.7%, 65.9%, 73.1%, and 78.2%, respectively.

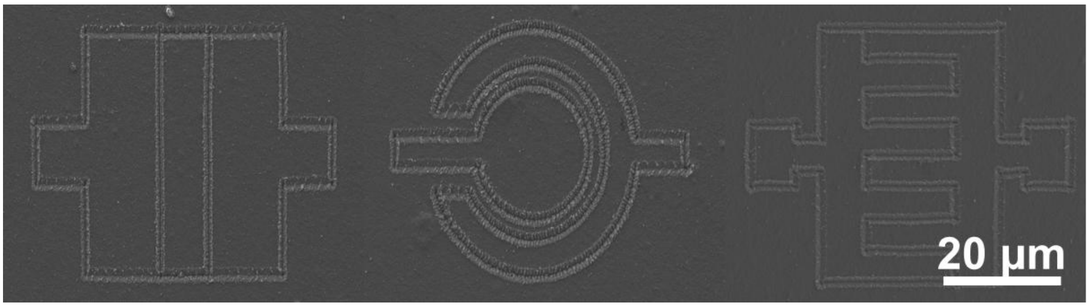

Supplementary Figure 23: SEM images of micro supercapacitors of different shapes (parallel strip, concentric circle and interdigital).

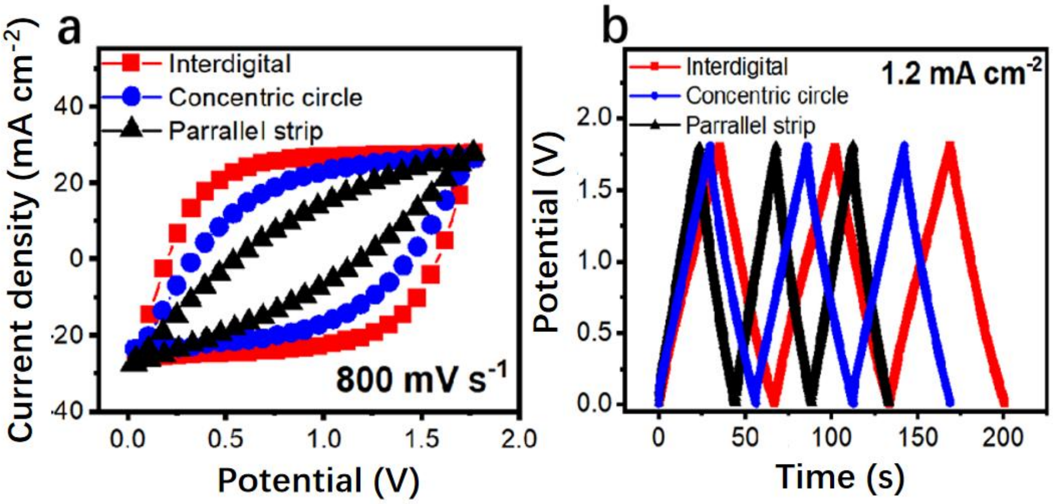

Supplementary Figure 24: a The CV curves of versatile-shaped MSCs obtained at a scan rate of 800 mV s^{-1}, **b** The GCD profiles of versatile-shaped MSCs tested at 1.2 mA cm^{-2}.

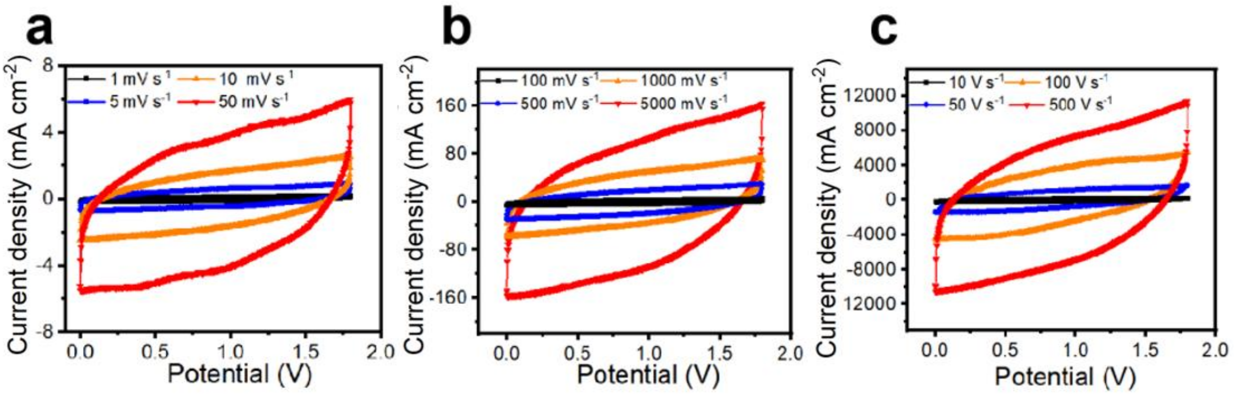

Supplementary Figure 25: **a-c** The CV curves of asymmetric MSCs prepared by our strategy at extremely high and very low scan rates. The regular rectangles indicate that asymmetric MSCs can retain good capacitance characteristics even at ultra-high scan rate.

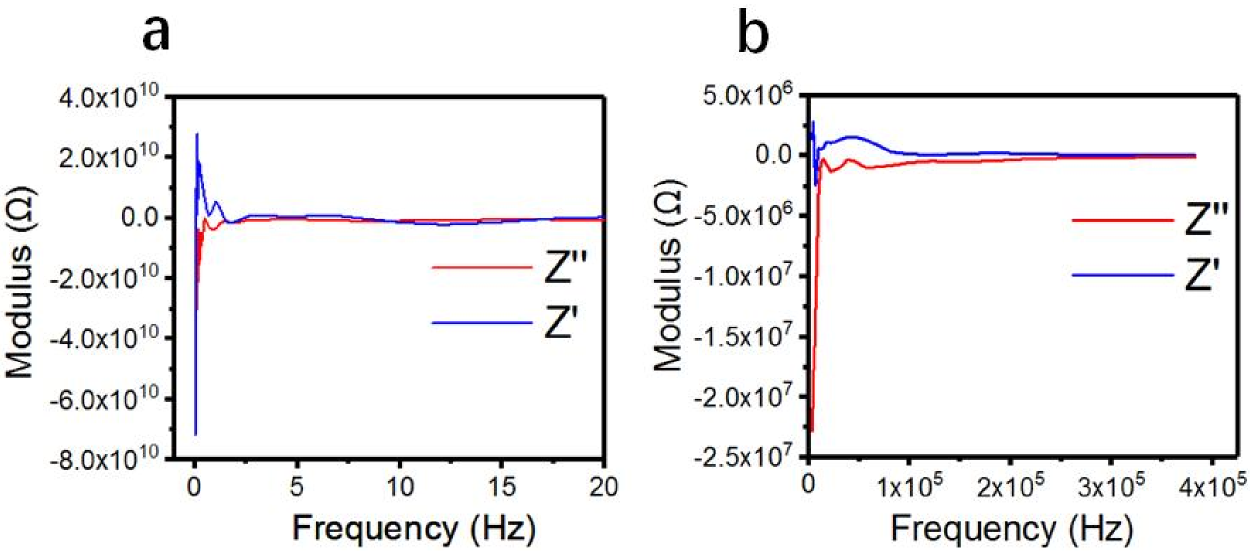

Supplementary Figure 26: The curve diagram of measured frequency and modulus changes at low (**a**) and high (**b**) frequencies by an impedance meter HP 4284 A.

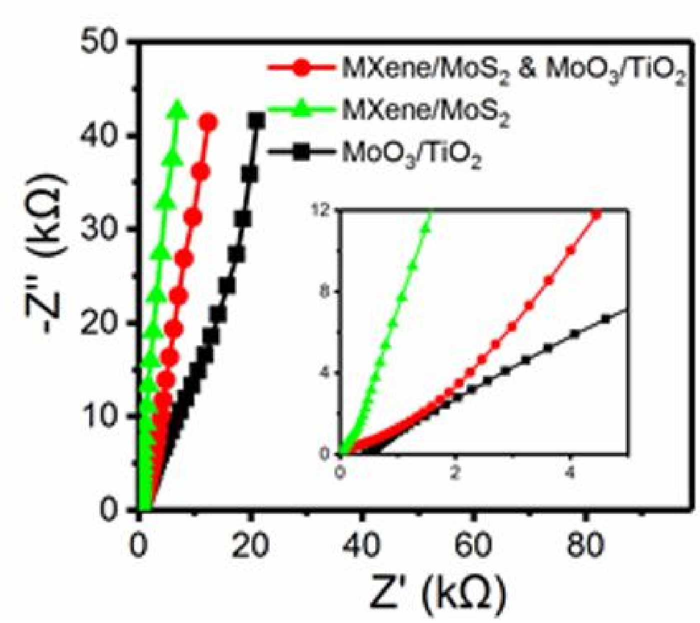

Supplementary Figure 27: Nyquist plots of the different types of MSCs (1T-MoS_2/MXene symmetric MSC, the 1T-MoS_2/MXene//MXene-derived TiO_2 and 1T-MoS_2-derived MoO_3 asymmetric MSC, and the laser-induced MXene-derived TiO_2 and 1T-MoS_2-derived MoO_3 symmetric MSC.

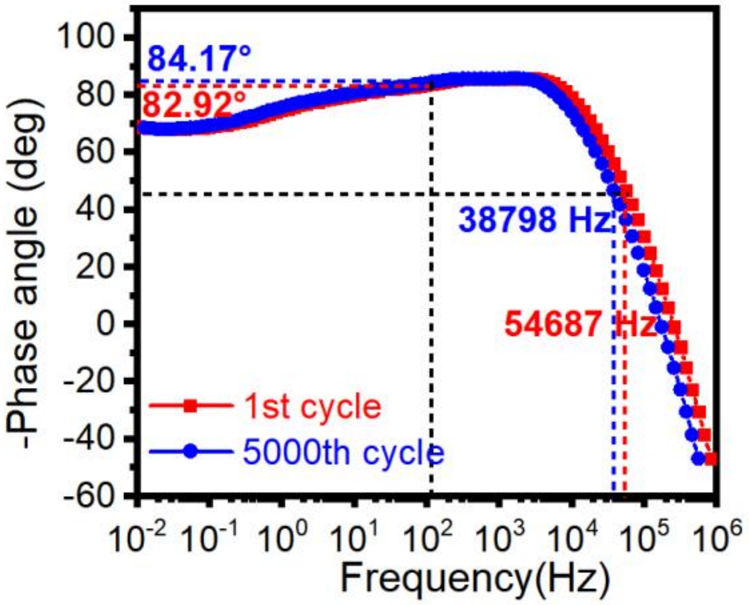

Supplementary Figure 28: The Bode phase angle plot of 1st cycle and 5000th cycles of the 1T-MoS_2/MXene//MXene-derived TiO_2 and 1T-MoS_2-derived MoO_3 asymmetric MSC.

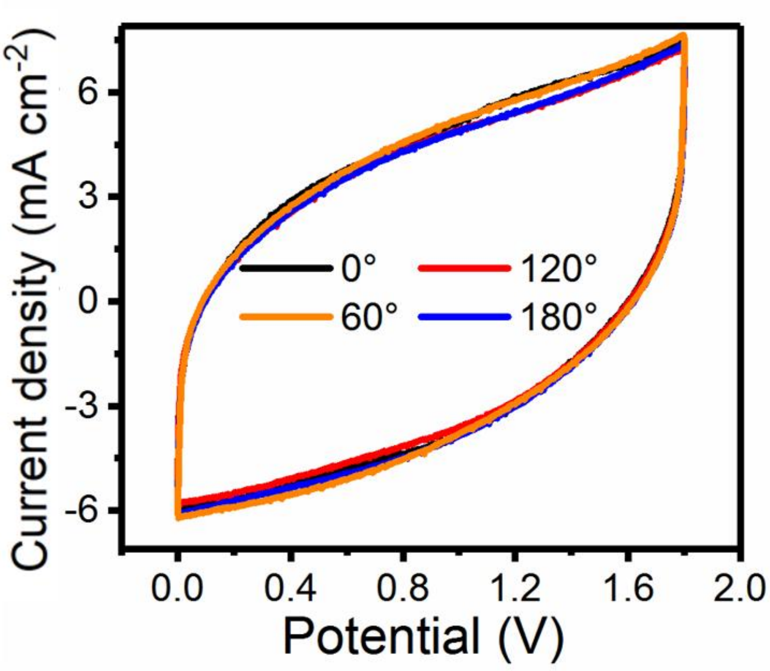

Supplementary Figure 29: Capacitance retention of the asymmetric MSCs obtained in different bending states compared with that in the flat state. Contrary to expectations, the CV curves are almost coincident. This outstanding flexibility will expand the use of our MSCs across numerous fields, including integrated circuits, wearable microelectronics, and medical devices.

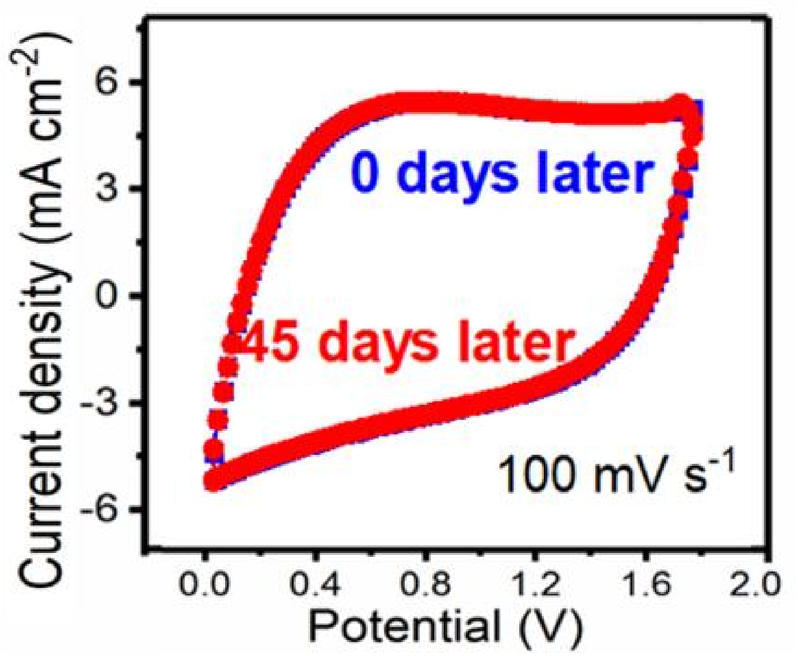

Supplementary Figure 30: CV curves of the fabricated micro-supercapacitors after 0 and 45 days.

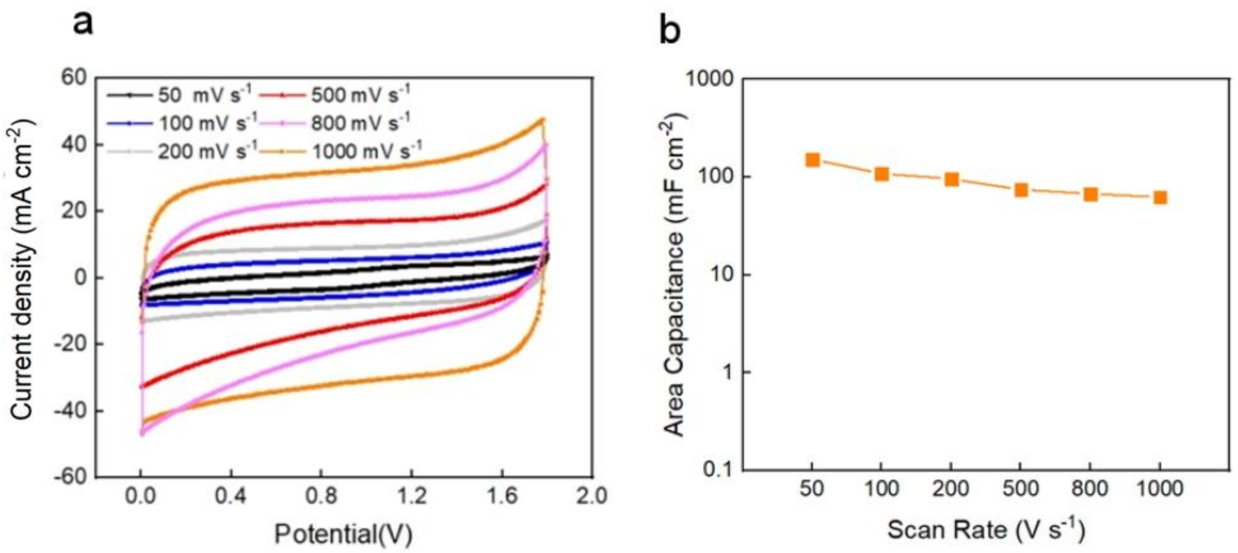

Supplementary Figure 31: a Cyclic voltammetry curves of the asymmetric MSCs under differing scan rates in the gel electrolyte. **b** Summary of the electrochemical performance of the MSCs under differing scan rates.

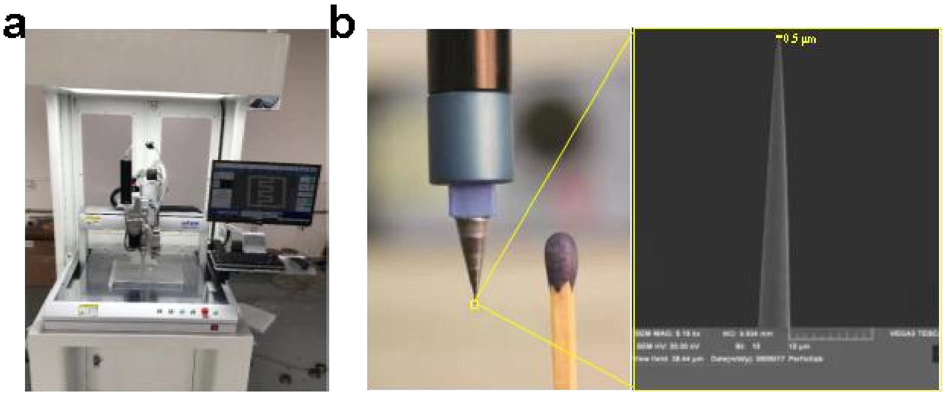

Supplementary Figure 32: (**a**) and (**b**) The figure of the dispensing machine with a confocal positioning system and the dispensing head with a precision needle of 0.5-micrometer diameter.

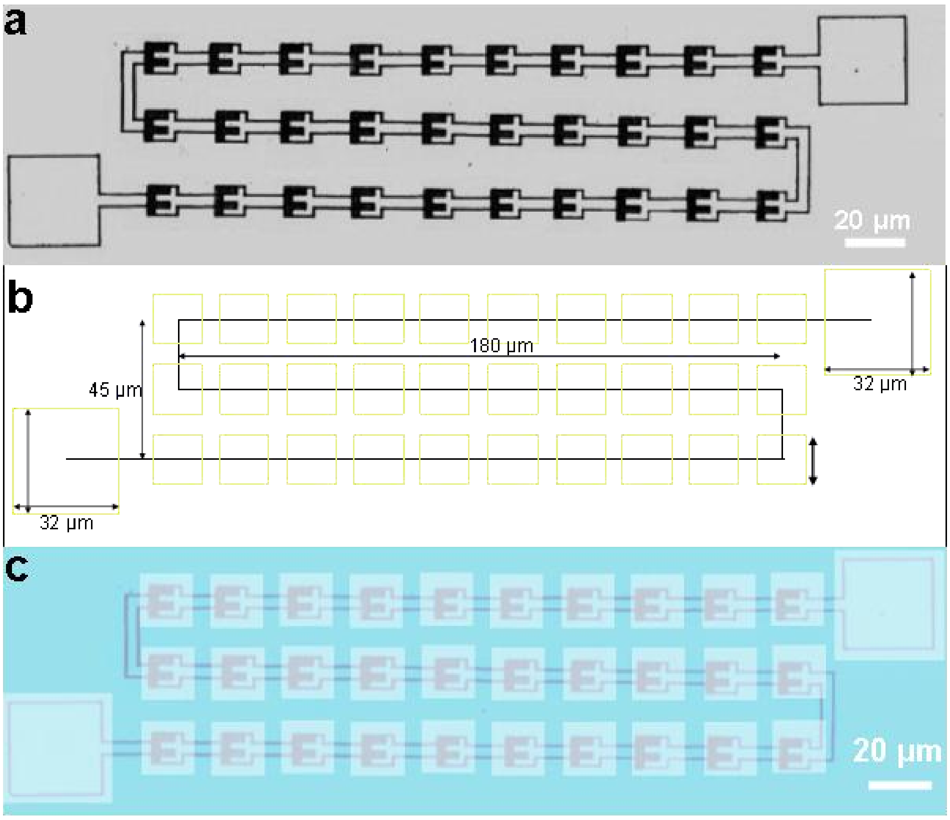

Supplementary Figure 33: **a** The image of 30 MSCs of 10 by 10 micron in series fabricated by TSSF. **b** The mask designed for photolithography. **c** The image of 30 MSCs of 10 by 10 micron in series after lithography.

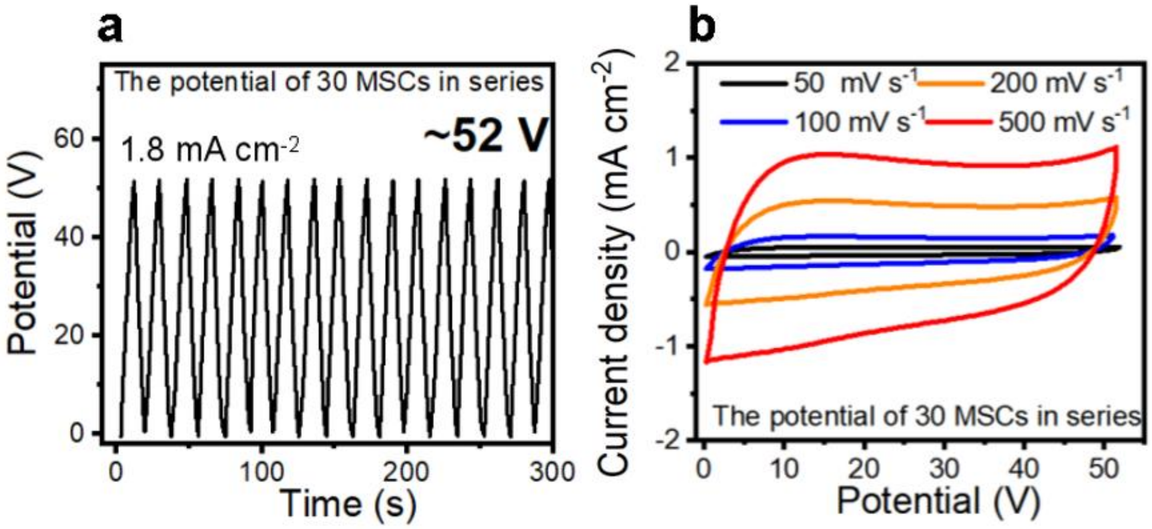

Supplementary Figure 34: (**a**) and (**b**) The galvanostatic charge-discharg profiles and cyclic voltammetry curves of the 30 integrated MSCs in series for the voltage window of 52 V.

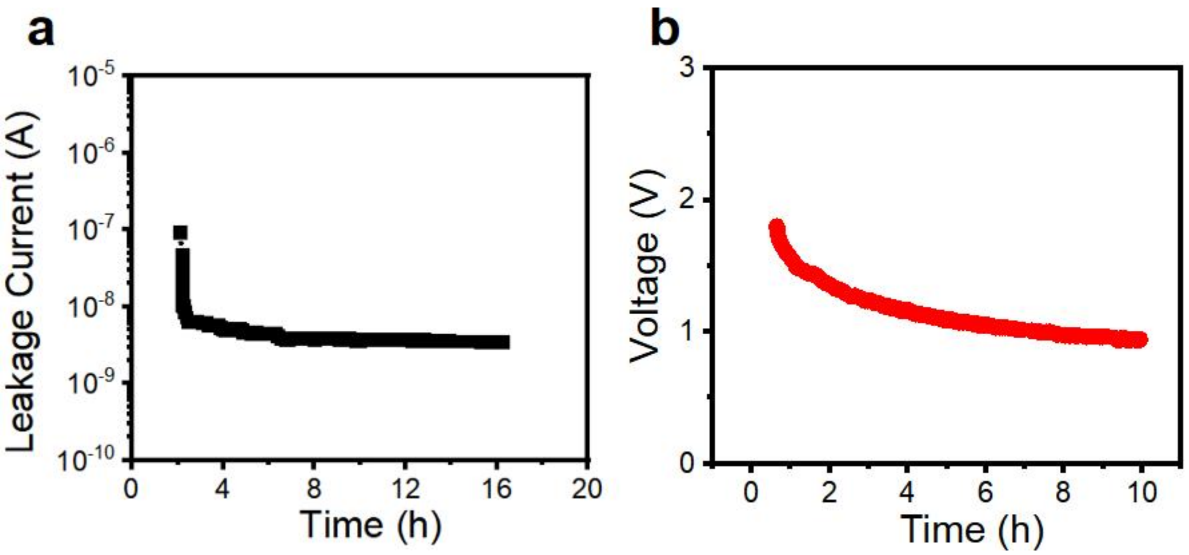

Supplementary Figure 35: a Leakage current measurement of our MSC and the current required to retain that voltage was measured over a period of 16 h. **b** Self-discharge curves of the respective MSC obtained immediately after recharging to V_{max}.

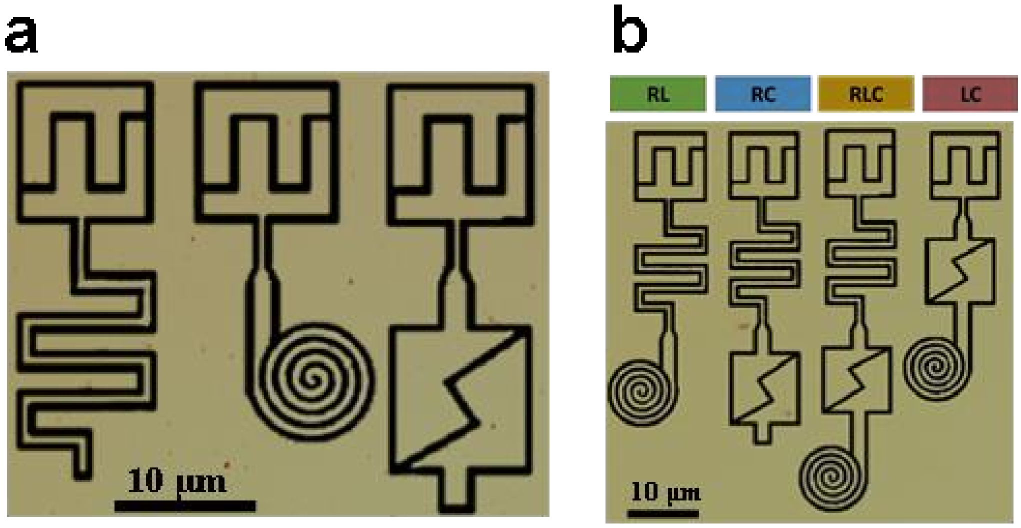

Supplementary Figure 36: a The integration of single interdigital MSC and another device for sensing by TSSL. **b** The fabrication of integrating MSCs into different logic circuits.

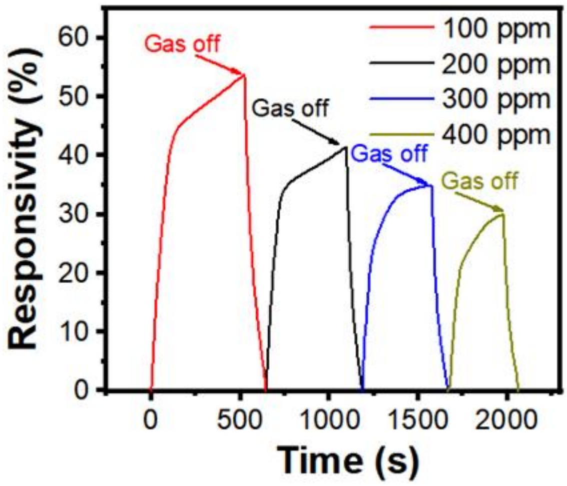

Supplementary Figure 37: The Gas-sensing responsivity under four different concentrations of ammonia gas (100, 200, 300, 400 ppm). To evaluate the sensing ability, two interconnected devices were employed to examine a single microsensing unit. The gas-sensitive response of the unit was measured at four different concentrations of ammonia (100, 200, 300, and 400 ppm), as shown in Figure R23. The gas sensor exhibited a responsiveness value of 54.23% at 100 ppm ammonia concentration, which increased as the concentration decreased. However, at 400 ppm, the response rate declined to 26.8%. The response and recovery times decreased with increasing gas concentrations.

Supplementary Table

Table 1. Comparison of asymmetric planar micro-supercapacitors prepared by different processes.

AMSCs	Machining technology	Size (mm)	Resolution (μm)	Refs
CuSe@FeOOH// CuSe@MnOOH	Alternate Electrodeposition	2×2	100	1
Mesoporous Fe_2O_3/MnO_2	Inkjet printing /Deposition	20×20	20	2
$Co(OH)_2$//erGO	Photolithography /Electrodeposition	4×4	10	3
$Ni(OH)_2$/Ag-NW //rGO/Ag-NW	Laser-Printing/ Coating	40×30	1000	4
PNG//PNG-MoO_2	Laser cutting /Reassembly	5×10	1000	5
LIG-FeOOH //LIG-MnO_2	Laser reduction /Electrodeposition	20×40	1000	6
Graphene quantum dots//PANI	Two-step electrodeposition	10×10	400	7
**1T-MoS_2/MXene//laser-induced MXenes-derived TiO_2 and 1T-MoS_2-derived MoO_3**	**Shaped femtosecond laser pulse patterning**	**0.01×0.01**	**0.2**	**Our work**

Table 2 Comparison of micro-supercapacitors prepared by laser with our strategy.

Materials	Types	Method	Capacitance ($mF\ cm^{-2}$)	Size (mm)/ Resolution(μm)	Manufacturing efficiency/30min	Reference
Laser-scribed graphene	Symmetric	Laser writing reduction	5.02	10×10 /-	1.5	8
Laser-irradiation graphene	Symmetric	Laser writing reduction	15.38	4×15 /-	2	9
$Ti_3C_2T_x$ MXenes	Symmetric	Spray-coating / Laser cutting	27.3	8×6 /109	-	10
MXene// Graphene	Asymmetric	Laser cutting/ Spray-coatin/ Laser cutting/ Spray-coating	2.4	12×8 /400	-	11
MXene	Symmetric	Laser etching	4.0	1×1 /10	5	12
TiO_2-coated graphene	Symmetric	Laser drilling	10.2	- /40	-	13
1T MoS_2	Symmetric	Laser etching	36	0.1×0.1 /0.83	100	14
MXene/1TMoS_2 //MoO_3/TiO_2	Asymmetric	Laser pattern	220	0.01×0.01 /0.2	90,000	Our Work
MXene/1TMoS_2	Symmetric	Laser pattern	98	0.01×0.01 /0.2	180,000	Our Work
MoO_3/TiO_2	Symmetric	Laser pattern	87	0.01×0.01 /0.2	60,000	Our Work

Table 3. Summary of the energy dispersive spectrometer of molybdenum oxide and titanium oxide for the different laser-induced TM.

Material	C	O	Mo	S	Ti
MT-10/0	52.15%	32.01%	4.34%	2.22%	9.28%
MT-10/10	49.19%	34.20%	4.12%	2.04%	10.45%
MT-20/0	24.83%	36.79%	9.94%	4.97%	23.47%
MT-20/10	18.86%	37.45%	11.43%	6.28%	25.98%
MT-30/0	17.18%	36.42%	14.76%	5.18%	27.55%
MT-30/10	11.94%	36.33%	13.45%	6.89%	31.39%

**Table 4. Comparison of the thickness and volumetric power density and energy density of miniature supercapacitors.**				
**Sample (electrode)**	**Thickness (μm)**	**Power density （W cm^{-3}）**	**Energy density （mW h cm^{-3}）**	**Reference**
rGO/MnO_2	1	136	230	15
CuSe@FeOOH//CuSe@MnOOH	0.8	129.9	160	16
FIB-rGO	0.6	5.1	210	17
Ni/MnO_2	5	80	4.7	18
LiPON/Co_3O_4	0.1	18	10	19
CNF PEDOT:PSS	2	13.4	0.34	20
**Our work**	**1**	**5581**	**495**	**Our work**

**Table 5. Comparison of areal power density and energy density of ultra thin miniature supercapacitors.**			
**Sample (electrode)**	**Power density （$mW\ cm^{-2}$）**	**Energy density （$\mu W\ h\ cm^{-2}$）**	**Reference**
PANI/RGO	0.29	19.78	21
N-doped CNT	10.31	8.23	22
rGO/Ti_3C_2 aerogel	0.31	2.51	23
MXene/graphene	0.06	3.18	24
$Ti_3C_2T_x$/CNF/PC	2.35	2.42	25
Graphene/MnO_2	0.48	29.61	26
CF-MnO_2/CF-MoO_3	8.12	1.99	27
MnO_2/OLC	0.06	0.33	28
**Our work**	**55.97**	**49.5**	**Our work**

Legends for supplementary movies

Supplementary movie 1 shows that the technology can be used to process symmetric patterned MSCs super quickly. The size of each MSC processed was $10\times10\ \mu m^2$.

Supplementary movie 2 shows that the technology can be used to process asymmetric patterned MSCs super quickly. The size of each MSC processed was $10\times10\ \mu m^2$.

Supplementary References

1. Li, J. C. et al. Alternate integration of vertically oriented CuSe@ FeOOH and CuSe@ MnOOH hybrid nanosheets frameworks for flexible in-plane asymmetric micro-supercapacitors. *ACS Appl. Energ. Mater*. **3,** 3692-3703 (2020).
2. Xia, Z. Y. et al. Selective deposition of metal oxide nanoflakes on graphene electrodes to obtain high-performance asymmetric micro-supercapacitors. *Nanoscale* 2021,**13,** 3285-3294 (2021).
3. Lee, S. et al. All-solid-state flexible asymmetric micro supercapacitors based on cobalt hydroxide and reduced graphene oxide electrodes. *RSC Adv.* **6,** 43844-43854 (2016).
4. Huang, G. W. et al. Laser-printed in-plane micro-supercapacitors: from symmetric to asymmetric structure. *ACS Appl. Mater.I interfaces* **10,** 723-732 (2018).
5. Zhang, L. Z. et al. Shape-tailorable high-energy asymmetric micro-supercapacitors based on plasma reduced and nitrogen-doped graphene oxide and MoO_2 nanoparticles. *J Mater. Chem. A* **7,** 14328-14336 (2019).
6. Sun, X. et al. Electrodeposited with FeOOH and MnO_2 on laser-induced graphene for multi-assembly supercapacitors. *J. Alloys Compd.* **893,** (2022).
7. Liu, W. et al. Novel and high-performance asymmetric micro-supercapacitors based on graphene quantum dots and polyaniline nanofibers. *Nanoscale,* **5,** 6053-6062 (2013).
8. El-Kady, M. F. et al. Laser scribing of high-performance and flexible graphene-based electrochemical capacitors. *Science,* **335,** 1326-1330 (2012).
9. Wu, M. et al. A high-performance current collector-free flexible in-plane micro-supercapacitor based on a highly conductive reduced graphene oxide film. *J Mater. Chem. A* ***4*****,** 16213-16218 (2016).
10. Peng, Y. Y. et al. All-MXene (2D titanium carbide) solid-state microsupercapacitors for on-chip energy storage. *Energy Environ. Sci.*, **9,** 2847-2854 (2016).
11. Couly, C. et al. Asymmetric flexible MXene-reduced graphene oxide micro-supercapacitor. *Adv. Electron. Mater*, **4,** 1700339 (2018).
12. Li, Q. et al. Femtosecond laser-etched mxene microsupercapacitors with double-side configuration via arbitrary on-and through-substrate connections. *Adv. Energy Mater*, **10,** 2000470 (2020).
13. Xu, C. et al. Femtosecond laser drilled micro-hole arrays in thick and dense 2D nanomaterial electrodes toward high volumetric capacity and rate performance. *J. Power Sources,* **492,** 229638 (2021).
14. Xu, C. et al. Miniaturized high-performance metallic 1T-Phase MoS_2 micro-supercapacitors fabricated by temporally shaped femtosecond pulses. *Nano Energy*, **67,** 104260 (2020).
15. Yuan, Y. et al. Laser photonic-reduction stamping for graphene-based micro-supercapacitors ultrafast fabrication. *Nat. Commun.* **11,** 6185 (2020).
16. J. C. et al. Alternate integration of vertically oriented CuSe@ FeOOH and CuSe@ MnOOH hybrid nanosheets frameworks for flexible in-plane asymmetric micro-supercapacitors. *ACS Appl. Energ. Mater.* **3,** 3692-3703 (2020).
17. Lobo, D. E. et al. Miniaturized supercapacitors: focused ion beam reduced graphene oxide supercapacitors with enhanced performance metrics. *Adv. Energy Mater,* **5,** 1500665 (2015).
18. Guo, K. et al. Hand-drawing patterned ultra-thin integrated electrodes for flexible micro supercapacitors. *Energy Storage Materials,* **11,** 144-151 (2018).
19. Göhlert, T. et al. Ultra-thin all-solid-state micro-supercapacitors with exceptional performance and device flexibility. *Nano Energy,* **33,** 387-392 (2017).

20. Say, M. G. et al. Spray-coated paper supercapacitors. *npj Flexible Electronics*, **4,** 1-7 (2020).
21. Wu, X. et al. Construction of microfluidic-oriented polyaniline nanorod arrays/graphene composite fibers for application in wearable micro-supercapacitors. *J Mater. Chem. A,* **6,** 8940-8946 (2018).
22. Dubal, D. et al. Synthetic approach from polypyrrole nanotubes to nitrogen doped pyrolyzed carbon nanotubes for asymmetric supercapacitors. *J. Power Sources,* **308,** 158-165 (2016).
23. Radha, N. et al. Binder free self-standing high performance supercapacitive electrode based on graphene/titanium carbide composite aerogel. *Appl. Surf. Sci*, **481,** 892-899 (2019).
24. Yue, Y. et al. Highly self-healable 3D microsupercapacitor with MXene-graphene composite aerogel. *ACS Nano,* **12,** 4224-4232 (2018).
25. Chen, W. et al. Mxene ($Ti_3C_2T_x$)/cellulose nanofiber/porous carbon film as free-standing electrode for ultrathin and flexible supercapacitors. *Chem. Eng. J.* **413,** 127524 (2021).
26. Ma, Z. et al. High Surface Area of Crystalline/Amorphous Ultrathin MnO_2 Nanosheets Electrode for High-performance Flexible Micro-supercapacitors. *J. Alloys Compd*, **3,** 166012 (2022).
27. Noh, J. et al. High performance asymmetric supercapacitor twisted from carbon fiber/MnO_2 and carbon fiber/MoO_3. *Carbon* **116,** 470-478 (2017).
28. Wang, Y. et al. Printed all-solid flexible microsupercapacitors: towards the general route for high energy storage devices. *Nanotechnology,* **25,** 094010 (2014).
