# Supplementary material for: Laser maskless fast patterning for multitype microsupercapacitors
Source: Nat Commun. 2023 Jul 5;14:3967. doi: 10.1038/s41467-023-39760-3 (PMC10322851; doi:10.1038/s41467-023-39760-3)
Supplement: Supplementary file 2 — Description of Additional Supplementary Files [file 41467_2023_39760_MOESM2_ESM.pdf]

Description of Additional Supplementary Files

File Name: Supplementary Movie 1
Description: shows that the technology can be used to process symmetric patterned MSCs super quickly. The size of each MSC processed was 10×10 μm^2.

File Name: Supplementary Movie 2
Description: shows that the technology can be used to process asymmetric patterned MSCs super quickly. The size of each MSC processed was 10×10 μm^2.
