# Supplementary material for: Laser maskless fast patterning for multitype microsupercapacitors
Source: Nat Commun. 2023 Jul 5;14:3967. doi: 10.1038/s41467-023-39760-3 (PMC10322851; doi:10.1038/s41467-023-39760-3)
Supplement: Supplementary file 5 — Reporting Summary [file 41467_2023_39760_MOESM5_ESM.pdf]

nature research

Corresponding author(s): Xin Li

Lasing Reporting Summary

Nature Research wishes to improve the reproducibility of the work that we publish. This form is intended for publication with all accepted papers reporting claims of lasing and provides structure for consistency and transparency in reporting. Some list items might not apply to an individual manuscript, but all fields must be completed for clarity.

For further information on Nature Research policies, including our data availability policy, see Authors & Referees.

Experimental design

Please check: are the following details reported in the manuscript?

1. Threshold

Item	Yes	No	Explanation
Plots of device output power versus pump power over a wide range of values indicating a clear threshold	☒	☐	In the Method section of the manuscript, the threshold range that the laser can produce is explained.

2. Linewidth narrowing

Item	Yes	No	Explanation
Plots of spectral power density for the emission at pump powers below, around, and above the lasing threshold, indicating a clear linewidth narrowing at threshold	☐	☒	Our work does not involve the study of relevant tests.
Resolution of the spectrometer used to make spectral measurements	☐	☒	Our work does not involve the study of relevant tests.

3. Coherent emission

Item	Yes	No	Explanation
Measurements of the coherence and/or polarization of the emission	☐	☒	Our Gaussian laser is generated by the amplification stage and does not require this test.

4. Beam spatial profile

Item	Yes	No	Explanation
Image and/or measurement of the spatial shape and profile of the emission, showing a well-defined beam above threshold	☒	☐	The spatial shape of the laser is described in the manuscript (FIG. 1) and illustrated in the text.

5. Operating conditions

Item	Yes	No	Explanation
Description of the laser and pumping conditions *Continuous-wave, pulsed, temperature of operation*	☒	☐	In the Method section of the manuscript, the threshold range that the laser can produce is explained.
Threshold values provided as density values (e.g. W cm^{-2} or J cm^{-2}) taking into account the area of the device	☐	☒	This was not the case in the experiment.

6. Alternative explanations

Item	Yes	No	Explanation
Reasoning as to why alternative explanations have been ruled out as responsible for the emission characteristics *e.g. amplified spontaneous, directional scattering; modification of fluorescence spectrum by the cavity*	☐	☒	This was not the case in the experiment.

7. Theoretical analysis

Item	Yes	No	Explanation
Theoretical analysis that ensures that the experimental values measured are realistic and reasonable *e.g. laser threshold, linewidth, cavity gain-loss, efficiency*	☒	☐	The analysis of femtosecond laser action and the model established in the manuscript support the experimental results.

8. Statistics

Item	Yes	No	Explanation
Number of devices fabricated and tested	☒	☐	The statistics of the various devices on the optical path in the experiment are mentioned in the manuscript.
Statistical analysis of the device performance and lifetime (time to failure)	☒	☐	In the manuscript we emphasize that the device has very high stability.
